# Targetable vulnerabilities in T- and NK-cell lymphomas identified through preclinical models

**DOI:** 10.1038/s41467-018-04356-9

**Published:** 2018-05-22

**Authors:** Samuel Y. Ng, Noriaki Yoshida, Amanda L. Christie, Mahmoud Ghandi, Neekesh V. Dharia, Joshua Dempster, Mark Murakami, Kay Shigemori, Sara N. Morrow, Alexandria Van Scoyk, Nicolas A. Cordero, Kristen E. Stevenson, Maneka Puligandla, Brian Haas, Christopher Lo, Robin Meyers, Galen Gao, Andrew Cherniack, Abner Louissaint, Valentina Nardi, Aaron R. Thorner, Henry Long, Xintao Qiu, Elizabeth A. Morgan, David M. Dorfman, Danilo Fiore, Julie Jang, Alan L. Epstein, Ahmet Dogan, Yanming Zhang, Steven M. Horwitz, Eric D. Jacobsen, Solimar Santiago, Jian-Guo Ren, Vincent Guerlavais, D. Allen Annis, Manuel Aivado, Mansoor N. Saleh, Amitkumar Mehta, Aviad Tsherniak, David Root, Francisca Vazquez, William C. Hahn, Giorgio Inghirami, Jon C. Aster, David M. Weinstock, Raphael Koch

**Affiliations:** 1 Department of Medical Oncology, Dana-Farber Cancer Institute, 450 Brookline Ave, Boston, MA 02215 USA; 2 Broad Institute of Harvard and MIT, 415 Main St, Cambridge, MA 02142 USA; 3 Department of Pediatric Oncology, Dana-Farber Cancer Institute and Boston Children’s Hospital, 450 Brookline Ave, Boston, MA 02215 USA; 4 Harvard Medical School, 25 Shattuck Street, Boston, MA 02115 USA; 5 Department of Biostatistics and Computational Biology, Dana-Farber Cancer Institute, 450 Brookline Ave, Boston, MA 02215 USA; 6 Department of Pathology, Massachusetts General Hospital, 55 Fruit Street, Boston, MA 02114 USA; 7 Center for Cancer Genome Discovery, Dana-Farber Cancer Institute, 450 Brookline Ave, Boston, MA 02215 USA; 8 Center for Functional Epigenetics, Dana-Farber Cancer Institute, 450 Brookline Ave, Boston, MA 02215 USA; 9 Department of Pathology, Brigham and Women’s Hospital, 75 Francis St, Boston, MA 02215 USA; 10 Department of Pathology, Weill Cornell Medical College, 525 East 68th Street, New York, NY 10065 USA; 11 Department of Pathology, Keck School of Medicine, University of Southern California, 1975 Zonal Ave, Los Angeles, CA 90033 USA; 12 Department of Pathology, Memorial Sloan-Kettering Cancer Center, 1275 York Avenue, New York, NY 10065 USA; 13 Department of Medicine, Memorial Sloan-Kettering Cancer Center, 1275 York Avenue, New York, NY 10065 USA; 14 Aileron Therapeutics Inc, 281 Albany Street, Cambridge, MA 02139 USA; 15 University of Alabama-Birmingham Comprehensive Cancer Center, 1824 6th Avenue South, Birmingham, AL 3523 USA; 16 Present Address: Department of Hematology and Medical Oncology, University Medical Center Göttingen, Robert-Koch Str. 40, 37075 Göttingen, Germany

## Abstract

T- and NK-cell lymphomas (TCL) are a heterogenous group of lymphoid malignancies with poor prognosis. In contrast to B-cell and myeloid malignancies, there are few preclinical models of TCLs, which has hampered the development of effective therapeutics. Here we establish and characterize preclinical models of TCL. We identify multiple vulnerabilities that are targetable with currently available agents (e.g., inhibitors of JAK2 or IKZF1) and demonstrate proof-of-principle for biomarker-driven therapies using patient-derived xenografts (PDXs). We show that MDM2 and MDMX are targetable vulnerabilities within *TP53*-wild-type TCLs. ALRN-6924, a stapled peptide that blocks interactions between p53 and both MDM2 and MDMX has potent in vitro activity and superior in vivo activity across 8 different PDX models compared to the standard-of-care agent romidepsin. ALRN-6924 induced a complete remission in a patient with *TP53*-wild-type angioimmunoblastic T-cell lymphoma, demonstrating the potential for rapid translation of discoveries from subtype-specific preclinical models.

## Abstract (web summary)

T- and NK-cell lymphomas (TCL) are a group of lymphoid malignancies characterized by poor prognosis, but the absence of appropriate pre-clinical models has hampered the development of effective therapies. Here the authors establish several pre-clinical models and identify vulnerabilities that could be further exploited to treat patients afflicted by these diseases.

## Introduction

T-cell lymphomas (TCLs) comprise approximately 10% of all non-Hodgkin lymphomas (NHLs) in Western countries. Two-thirds of these lymphomas are peripheral TCLs (PTCLs) and the remainder are cutaneous TCLs (CTCLs). Many patients with CTCL have an indolent course that can span decades. In contrast, nearly all PTCLs are aggressive lymphomas and include both αβ and γδ T-cell lymphomas^1^. Emblematic of our poor understanding of PTCL biology, the most common subtype of PTCL is PTCL-NOS (not otherwise specified).

Treatment of PTCLs to date has been largely derivative or empiric. CHOP-based regimens, which were adopted based on their activity in B-cell lymphomas, remain the standard for first-line therapy. Except for ALK-rearranged anaplastic large-cell lymphoma (ALK+ ALCL), over 75% of PTCLs fail to respond or relapse after first-line therapy in <2 years. PTCL-NOS, angioimmunoblastic T-cell lymphoma (AITL), ALK-negative (ALK−) ALCL, and NK-TCL, the most common subtypes of PTCL, are each associated with <35% 5-year overall survival^2^. Long-term survivors of some like hepatosplenic T-cell lymphoma (HS-TCL) are extremely uncommon^2^.

Current approaches to treat PTCL are simply inadequate. The National Comprehensive Cancer Network guidelines list clinical trial as the top choice for all patients with PTCL (other than ALK+ ALCL) in both the first-line and relapsed/refractory settings. Improved understanding of the pathobiology of TCLs could highlight therapeutic targets, but there is a severe lack of well-characterized cellular and in vivo models of these lymphomas. To address this, we here report a comprehensive characterization of a newly established panel of patient-derived xenografts (PDXs) from multiple subtypes of TCL and available TCL cell lines. We highlight multiple potentially targetable genetic alterations and functional vulnerabilities. In *TP53*-wild-type TCL, MDM2, and MDMX are prominent therapeutic targets. ALRN-6924 shows promising pre-clinical activity in *TP53*-wt cell lines and PDX models and induced a complete remission in a patient with AITL. Thus, we demonstrate the potential of pre-clinical target validation and drug testing in models of rare cancers with direct clinical translation.

## Results

### Comprehensive characterization of TCL models

We collected a panel of previously established cell lines of T- and NK-cell lymphomas and validated their immunophenotype and STR-profiles (Supplementary Table 1, Supplementary Table 2). Furthermore, we defined the immunophenotypes of 17 TCL PDX models by flow cytometry and immunohistochemistry (IHC) and confirmed the expected histologic and phenotypic characteristics (Supplementary Table 2, Supplementary Fig. 1). To mitigate genetic drift that may occur over serial passaging^3^, we utilized PDXs in passage ≤2 and made these passages and affiliated data available through the Public Repository of Xenografts (www.PRoXe.org)^4^.

**Fig. 1:**
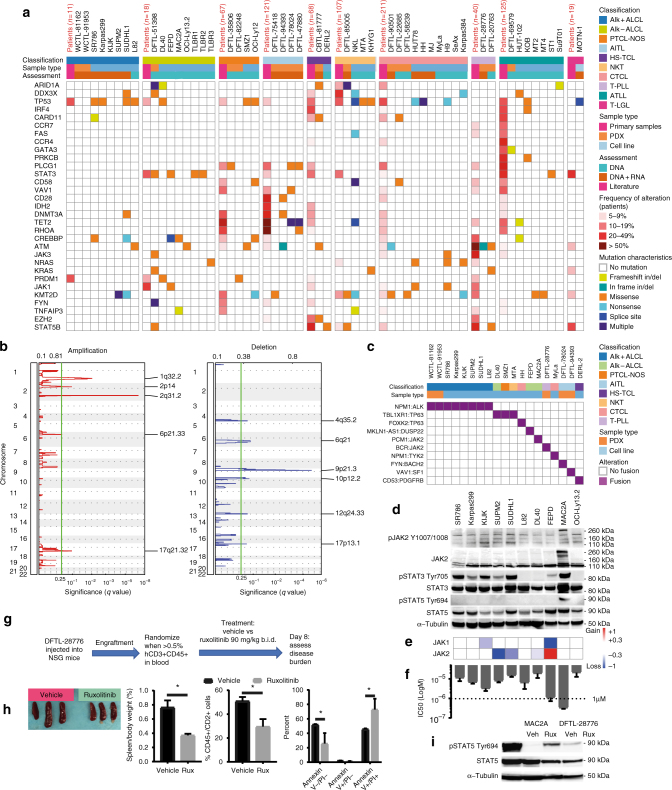
Genetic landscapes of TCL models identify targetable vulnerabilities. **a** Mutational characteristics of the most recurrently altered genes in patients, PDX models and cell lines of different subtypes of T- and NK-cell lymphomas. Highlighted in red is the frequency of recurrent mutations in different subtypes of T- and NK-cell lymphomas, based on published sequencing studies (Supplementary Table 3) with *n* indicating the number of cases included. **b** GISTIC summary plots of the significant copy number gains (left panel, red) and losses (right panel, blue) in 20 T-cell lymphoma cell lines. Y-axis: chromosomal position, *X*-axis: false-discovery rate (FDR) *q* values. **c** A subset of selected fusions in PDX models and cell lines, identified by RNA-seq. **d** Immunoblotting in ALCL cell lines for the indicated targets. Wild-type JAK2 is approximately 130 kDa and PCM-JAK2 is approximately 260 kDa. **e** Copy number variants of JAK1 and JAK2 corresponding to **d**. **f** IC50 values for ruxolitinib corresponding to **d**. **g** Workflow of the ruxolitinib in vivo trial. **h** Spleens were harvested from mice treated with vehicle or ruxolitinib. Spleen weight, infiltration of the spleens by hCD45/hCD2^+^ cells and AnnexinV/7-AAD staining of vehicle vs ruxolitinib treated mice. Statistics: Unpaired two-sided *t*-test, * *p* < 0.05. **i** Immunoblotting of MAC2A cells treated in vitro with DMSO or 1 μM ruxolitinib for 24 h and DFTL-28776 treated in vivo with vehicle or ruxolitinib for 7 days. Bar graphs in **f** and **i** indicate mean values of at least two independent experiments with error bars indicating standard error of the mean

To characterize genomic alterations across TCL models, we performed whole exome and transcriptome sequencing (RNA-seq) of 20 TCL lines using the algorithm established for the Cancer Cell Line Encyclopedia (https://portals.broadinstitute.org/ccle). We also sequenced 17 TCL PDX models (15 disseminated/orthotopic, 2 subcutaneous) and 13 additional cell lines using RNA-seq and targeted sequencing panels for recurrently mutated genes (Fig. 1a, Supplementary Tables 3, 4 and 5). Mutations identified in models derived from individual PTCL subtypes largely recapitulated those observed in patients (Fig. 1a, Supplementary Fig. 2). For example, ALK- ALCL frequently harbor *JAK1* and/or *STAT3* mutations^5^; these were also observed in TCL models (Fig. 1a), including 3 of 4 breast implant-associated ALCL models.

AITL is the second most-common subtype of PTCL but there were previously no cell lines or faithful in vivo models available. We generated four AITL PDXs, including models that harbor mutations in the most commonly mutated genes in this disease (*TET2, DNMT3A*, and/or *RHOA*) (Fig. 1a)^6, 7^. Similarly, no in vivo models of T-cell prolymphocytic leukemia (T-PLL) were previously available. We created two T-PLL PDXs, and confirmed *ATM* mutations and *TCL1* rearrangement that characterize this disease (Fig. 1a, Supplementary Fig. 1f).

To identify copy number alterations, we applied the GISTIC (Genomic Identification of Significant Targets in Cancer) algorithm to whole exome sequencing data from 20 cell lines. We identified recurrent amplifications and deletions, including deletions of 9p21.3 that include *CDKN2A* and 17p13.1 that include *TP53* (Fig. 1b, Supplementary Data 1). In fact, *TP53* was deleted and/or mutated in 15 of 21 (71.4%) TCL lines, whereas *TP53* alterations occur in <20% of patients with untreated PTCL or CTCL^6–8^. Of note, none of the 17 PDX models had *TP53* mutations (*p* < 0.001 compared to cell lines, Fisher’s exact test).

We utilized RNA-Seq to identify structural variants in TCL cell lines and PDXs. Recent studies have reported fusions involving *DUSP22* or *TP63* as recurrent events of prognostic significance in ALCL lacking *ALK* rearrangements^9^. RNA-Seq identified a *DUSP22* fusion in the FEPD cell line and fusions of *TP63* in 4 lines (Fig. 1c). The latter included TBL1XR1-TP63 and FOXK2-TP63 fusions, which were confirmed by sequencing and fluorescence in situ hybridization (Supplementary Fig. 3a–c). In all *TP63* fusions, the N-terminus of TP63 is deleted, creating a TP63 element similar to the ΔNTP63 (p40) variant (Supplementary Fig. 3a)^10^.

Multiple additional potentially targetable fusions were identified in cell lines or PDX models (Supplementary Fig. 3d, Supplementary Data 2, Supplementary Table 6), including NPM1-TYK2 in MyLa CTCL cells^11^, CD53-PDGFRB in DERL-2 HS-TCL cells, and VAV1-SF1 in an AITL PDX (Fig. 1c)^12^. We noted fusions that maintained the kinase domain of JAK2 in both MAC2A ALCL cells^13^ and the T-PLL PDX DFTL-28776 (Fig. 1c). Immunoblotting of MAC2A cells demonstrated constitutive phosphorylation of STAT3, STAT5, and PCM1-JAK2 (Fig. 1d). MAC2A cells lacked copy number alterations at *JAK2* (Fig. 1e) and were highly sensitive to ruxolitinib, an FDA-approved JAK1/JAK2 inhibitor (Fig. 1f). We engrafted mice with DFTL-28776 and treated for 7 days with ruxolitinib or vehicle (Fig. 1g). Spleen size and T-PLL involvement were markedly reduced in ruxolitinib-treated mice. Residual T-PLL cells had decreased STAT5 phosphorylation and were >70% apoptotic (Fig. 1h, i).

To characterize the transcriptomes of TCL cell lines and PDXs, we applied a sample–sample correlation matrix to cluster gene expression profiles across models. Both ALK+ ALCL and AITL formed distinct clusters, consistent with their unique cells-of-origin, while the remaining models formed a heterogeneous cluster (Supplementary Fig. 4a). As expected, gene set enrichment analysis (GSEA) identified enrichment of STAT3 target genes in ALCL models and enrichment of follicular helper T-cell (T_FH_) genes in AITL models (Supplementary Fig. 4b, c and Supplementary Data 3 and 4).

### CRISPR-Cas9 screen identifies vulnerabilities in TCL

**Fig. 2:**
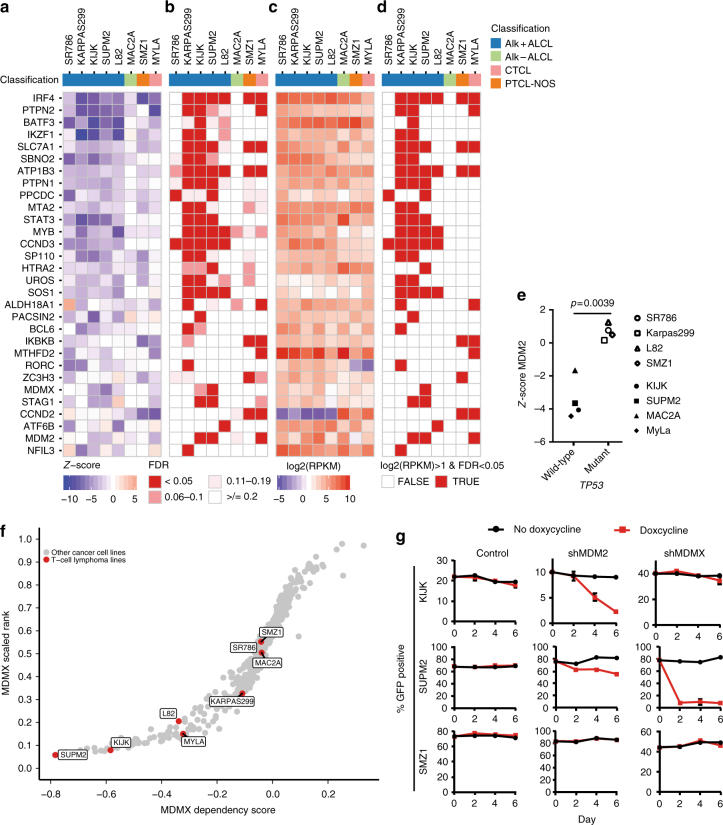
Genome-scale vulnerability screens reveal unique and common targets. **a** T-cell lymphoma specific vulnerabilities, ranked by *Z*-score and recurrence. **b** Corresponding false-discovery rate (FDR) *q*-values. **c** Corresponding gene expression level; RPKM, reads per kilobase of transcript, per million mapped reads. **d** Combined gene expression level and FDR. **e** Dependency score of MDM2 in *TP53* -wild-type versus *TP53*-mutated cell lines. Comparison is by two-sided *t*-test with Welch correction. **f** MDMX dependency score across 391 cancer cell lines with TCL lines highlighted in red. **g** Knockdown of MDM2 and MDMX in *TP53*-wild-type cell lines KI-JK and SUP-M2 and *TP53*-mutated cell line SMZ-1 using GFP-expressing, doxycycline inducible shRNA. Data points indicate mean values with error bars indicating standard error of the mean

To begin defining additional vulnerabilities across TCL subtypes, we utilized a genome-scale CRISPR-Cas9 loss-of-function screen. Of 21 lines, 8 (representing 4 subtypes) were adequately transducible with sufficient Cas9 activity for screening. We ranked T-cell lymphoma-specific dependencies by comparing to 383 additional lines screened using the same platform^14^ (Supplementary Data 5 and 6). Integration of gene expression, recurrence and statistical significance (FDR *q*-value <0.05) resulted in a list of 30 genes as top vulnerabilities (Fig. 2a–d). *IRF4*, *STAT3*, and *BCL6* were previously implicated as vulnerabilities in ALCL^5, 15^ but the remaining genes are largely novel discoveries for TCL.

We validated IRF4 dependence across multiple cell lines with shRNA (Supplementary Fig. 5a). IRF4 is known to be regulated by the IKAROS Family Zinc Finger 1 (IKZF1) transcription factor, which was also a vulnerability across multiple lines (Fig. 2a–d). IRF4 was previously identified as a vulnerability in ALCL, where it is positively regulated by CD30^16^ and STAT3^17^. Our data demonstrate that cell lines from both PTCL-NOS and CTCL subtypes are also dependent on IRF4. In B-cell malignancies, lenalidomide is known to induce IKZF1 and IKZF3 degradation by acting as a proteolysis-targeting chimera (PROTAC)^18^. Indeed, lenalidomide treatment reduced proliferation in 4 of 7 tested cell lines (Supplementary Fig. 5b). A preliminary analysis demonstrated lower IRF4 protein levels in lines that were sensitive to lenalidomide (*p* = 0.0025, unpaired two-sided *t*-test; Supplementary Fig. 5c, d). A larger analysis of primary specimens will be needed to determine whether low IRF4 protein level could serve as a predictive biomarker for lenalidomide response in patients with TCL.

We noted that the phosphatase PTPN2 was the 2nd ranked vulnerability across lines. PTPN2 is an important regulator of T-cell tolerance and development^19^ and PTPN2 loss in tumor cells can enhance sensitivity to immune checkpoint blockade^20^. Confirming the results of the CRISPR-CAS9 screen, we validated PTPN2 as a specific vulnerability in KI-JK cells but not in SMZ-1 cells, which were not sensitive to PTPN2 knockout in the screen and in an independent validation (Supplementary Fig. 5e, f).

All 4 ALK+ ALCL lines tested in the CRISPR-CAS9 screen were sensitive to the ALK inhibitor crizotinib but the ALK- ALCL line FEPD was not. We noted that ALK did not score as a vulnerability in any of the ALK+ ALCL lines. This was expected as all 4 sgRNAs targeting *ALK* in the CRISPR-CAS9 screen were directed against the 5′ portion of the gene that is not included in the NPM-ALK fusion (Supplementary Fig. 5g, h).

### MDM2 and MDMX are targetable vulnerabilities in *TP53*-wild-type TCL

**Fig. 3:**
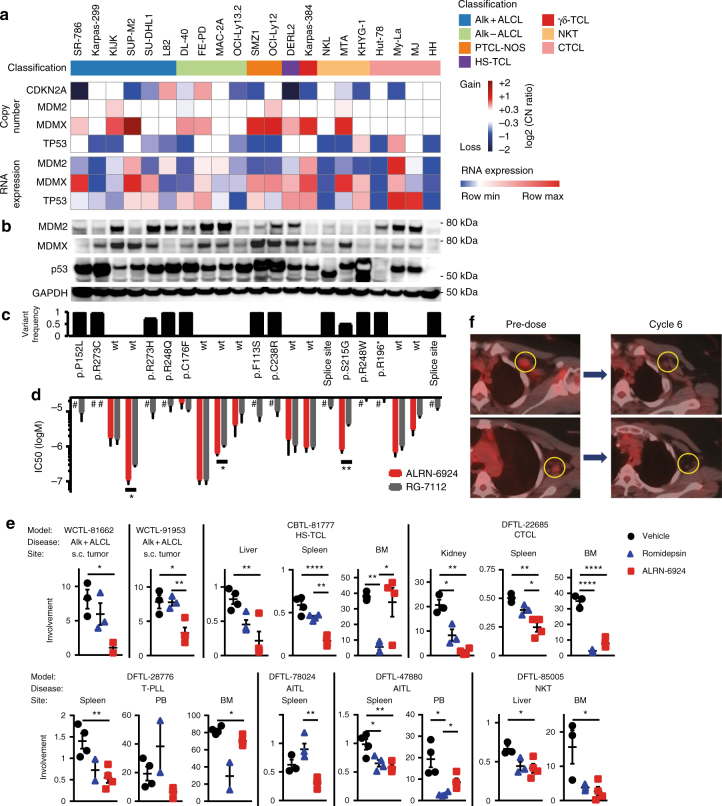
Targeting MDMX and MDM2 with ALRN-6924 **a** Copy number variants and RNA expression levels of *MDM2*, *MDMX*, and *TP53* across 21 T- and NK-cell lymphoma cell lines. **b** Protein levels of MDM2, MDMX and p53 by immunoblotting, corresponding to **a**. **c** *TP53* mutation status, corresponding to **a**. **d** IC50 values of ALRN-6924 and RG-7112, corresponding to **a**. Bar graphs indicate mean values of at least two independent experiments performed in quadruplicates with error bars indicating standard error of the mean. Statistics: Unpaired two-sided *t*-test, *p < 0.05, **p < 0.01. **e** Tumor burden at all involved sites for each mouse. Tumor involvement is represented as % tumor weight per body weight for subcutaneous (s.c.) tumors, % spleen per body weight and % infiltration of hCD45/hCD2^+^ cells for bone marrow (BM) and peripheral blood (PB). Liver involvement is presented in grams, assessed by liver weight x % infiltration of hCD45/hCD2^+^ cells. Comparisons are by two-sided *t*-test with Welch correction. Error bars indicate standard error of the mean. **f** PET-CT scan showing axillary lymph node involvement with AITL at two sites (circled) prior to treatment with ALRN-6924 and complete remission after cycle 6

We noted that both MDM2 and MDMX, which negatively regulate wild-type p53 function, scored as vulnerabilities in the CRISPR-Cas9 screen exclusively in the 4 *TP53-*wild-type TCL lines (Fig. 2a–e, Supplementary Fig. 6a). Notably, the SUP-M2 ALCL line harbors an *MDMX* gene amplification with high protein expression (Fig. 3a, b) and had the highest dependency score for MDMX among the 391 cell lines screened (Fig. 2f). We validated functional dependences on both MDM2 and MDMX using doxycycline-inducible shRNA (Fig. 2g).

To inhibit MDM2 and MDMX, we utilized the stapled peptide ALRN-6924, which is currently in phase I/II trials^21^. ALRN-6924 was potently active against all 9 *TP53*-wild-type cell lines (IC50 100 nM–4 μM) and in MTA cells, which harbor a heterozygous S215G mutation that is believed to confer loss-of-function. In contrast, all 11 lines with homozygous, hemizygous or dominant negative (p.R273H^22^ in SUDHL1) *TP53* mutations had IC50 values >10 μM (Fig. 3c, d). In *TP53*-wild-type lines, ALRN-6924 induced a dose-dependent increase in levels of both p53 protein and the p53-target p21, which was associated with G0/G1 cell cycle arrest and induction of apoptosis (Supplementary Fig. 6c–e). ALRN-6924 had superior potency to the small molecule MDM2 inhibitor RG-7112 in a subset of lines, including 2 lines with *MDMX* amplification (Fig. 3a,d, Supplementary Fig. 6f–h).

Because of the broad activity of ALRN-6924, we assessed its efficacy in eight *TP53*-wild-type PDX models representing six different TCL subtypes. We established a maximum tolerated dose (MTD) for the standard-of-care agent romidepsin of 1 mg per kg given intraperitoneally on days 1, 4, and 7 (Supplementary Fig. 7a), which was the same dose previously used in lymphoma cell line xenografts^23^. We compared romidepsin at this dose to ALRN-6924 dosed at 20 mg per kg given intravenously on days 1, 4, and 7. This dose of ALRN-6924 results in a similar area under the plasma concentration curve (AUC, 686–759 µg x h x mL^−1^) in mice to the AUC in patients (831 µg x h x mL^−1^) after a single dose of 3.1 mg per kg^21^, which is the recommended phase II dose.

Mice were xenografted with tumor, allowed to develop significant disease burden and then randomized to treatment with vehicle, romidepsin or ALRN-6924 on days 1, 4 and 7. All mice were sacrificed on day 8 to assess disease burden across compartments. ALRN-6924 treatment induced p21 expression by IHC and apoptosis in treated tumors (Supplementary Fig. 6i, j). ALRN-6924 was broadly active across bone marrow, spleen and other involved compartments in all 8 models. ALRN-6924 was superior or equivalent to romidepsin across models, with the exception of bone marrow disease in 2 models (Fig. 3e).

In the phase 1/2a trial of ALRN-6924 (NCT02264613), the first patient treated for TCL experienced a complete remission (Fig. 3f). She was diagnosed with AITL and relapsed almost immediately after first-line CHOEP chemotherapy. A repeat biopsy confirmed AITL with wild-type *TP53* and no copy number alterations at *TP53, MDM2*, or *MDMX*. She was started on ALRN-6924 given weekly for 3 weeks out of 4. She remains in a complete remission after two years of therapy.

## Discussion

We established and comprehensively characterized preclinical models of TCL to facilitate both mechanistic discovery and biomarker-driven therapy. Immunophenotyping, DNA sequencing and RNA-based clustering all suggest that these models recapitulate important aspects of TCL biology. Importantly, the PDX and cell line models provide a pre-clinical toolbox for interrogating genetic, epigenetic, metabolic and other alterations of potential clinical significance.

Our CRISPR-CAS9 screen identified multiple new targets but was limited by the transducibility of available cell lines. Clearly, additional efforts are needed to extend genome-wide vulnerability screening to additional lines from other subtypes. In the 8 lines we screened, we rediscovered multiple known targets, most notably IRF4. It remains unclear whether clinical responses to lenalidomide, which occur in approximately 22% of patients with relapsed/refractory PTCL^24^, involve the degradation of IKZF1, as shown in B-cell malignancies^18^. Further studies are needed to clarify whether responses to lenalidomide among patients with PTCL result from tumor cell-autonomous responses and, if so, to identify the relevant targets that are degraded in the presence of lenalidomide.

We were surprised that PTPN2 scored as a vulnerability in the CRISPR-CAS9 screen in 7 of 8 cell lines. PTPN2 was previously described as a tumor suppressor in T-ALL^25, 26^. PTPN2 can negatively regulate JAK/STAT signaling in CD8+ T-cells^27^. In fact, deletion of PTPN2 in mature T cells results in autoimmunity in aged mice, possibly due to a decreased threshold for T-cell receptor (TCR) signaling^28^. A previous analysis identified biallelic frameshift mutations in one of 69 PTCLs^29^, suggesting that PTPN2 could also function as a tumor suppressor in mature T cell malignancies. An alternate possibility is that hyperactivation of TCR or JAK/STAT signaling resulting from PTPN2 loss (or inhibition) is toxic to some or all mature T cells. This concept was previously demonstrated for multiple phosphatases that buffer B-cell receptor signaling in B-cell malignancies^30^. Further studies are needed to determine the role of PTPN2 in PTCLs and whether loss of this phosphatase can induce a central tolerance checkpoint in lymphoma cells^31^.

We validated JAK2 fusions and wild-type p53 as targets for small molecule inhibitors using in vivo PDX models. The presence of a JAK2 fusion in a T-PLL PDX was unexpected, but aberrant activation of this pathway is consistent with the activating JAK/STAT pathway mutations that have been described in T-PLL^32^. The in vivo response that we observed suggests that identifying JAK2 fusions in this disease could offer significant clinical benefit, especially in a disease like T-PLL with no standard cytotoxic or targeted regimens for second-line therapy.

We showed that ALRN-6924 has broad activity against multiple TCL lines and PDXs. It is extremely complicated to compare the activity of multiple drugs across multiple models, especially because the pharmacokinetics of each drug differ between mouse and human. Nonetheless, we used a maximum tolerated dose of romidepsin as a comparator to provide some relevance to current clinical treatment. The data across all 8 lines suggest that romidepsin has impressive activity in the bone marrow but ALRN-6924 was active across nearly all involved sites. Further studies are needed to understand whether these differences in compartment-specific activity are related to drug distribution or other microenvironmental determinants of efficacy.

Our study demonstrates that data from genome-wide vulnerability screens and in vivo PDX trials are highly complementary and can form the basis for clinical testing in rare cancers. We continue to generate models, including two additional AITL PDXs and one additional PTCL-NOS PDX that engrafted during the review of this manuscript. All models are available to both academic and industry investigators and will hopefully facilitate the development of more effective treatment strategies for patients with TCL.

## Methods

### Cell lines, antibodies, and small molecules

SR-786^33^, KI-JK^34^, SUP-M2^35^, SU-DHL-1^36^, L82^37^, DERL2^38^, and MOTN-1^39^ cells were obtained from DSMZ, Karpas 299^40^, Karpas 384^41^, MyLa^42^ cells from Sigma-Aldrich, HUT78^43^, MJ^44^ from ATCC, and DL-40^45^, MTA^46^, and KHYG-1^47^ cells from JCRB. TLBR1^48^, TLBR2^49^ and TLBR3^49^ were kindly provided by Alan L. Epstein (Keck School of Medicine, University of Southern California, Los Angeles, USA). FEPD^50^, MAC2A^51^, OCI-Ly13.2^52^ and OCI-Ly12^52^ were kindly provided by Leandro Cerchietti (Weill Cornell Medicine, New York, USA as approved by the Ontario Cancer Institute) and NKL^53^ cells were kindly provided by Jerome Ritz (Dana-Farber Cancer Institute, Boston, USA). HH^54^, H9^43^, and SeAx^55^ were kindly provided by Anthony G. Letai (Dana-Farber Cancer Institute, Boston, USA). HUT102^56^, MT2^57^, and MT4^58^ were kindly provided by Koichi Ohshima (Kurume University School of Medicine, Kurume, Japan). KOB^59^ and ST1^59^ were kindly provided by Yasuaki Yamada (Nagasaki University School of Medicine, Nagasaki, Japan), Su9T01^60^ was kindly provided by Naomichi Arima (Kagoshima University School of Medicine, Kagoshima, Japan), and SMZ-1^61^ cells were kindly provided by Hitoshi Ohno (Tenri Medical Institute, Tenri, Japan).

Cells were cultured according to the vendor’s recommendation. Cell lines were routinely tested for mycoplasma (ATCC® Universal Mycoplasma Detection Kit) and authenticity was validated by short tandem repeat (STR)-profiling.

### Drugs

Ruxolitinib, RG-7112, RG-7388, and romidepsin were purchased from Selleckchem. ALRN-6924 was provided by Aileron Therapeutics.

### Proliferation, cell cycle, and apoptosis assays

Proliferation assays with ruxolitinib and ALRN-6924 were performed in a 384-well format, using a JANUS Automated Workstation (PerkinElmer) and CellTiter-Glo Luminescent Cell Viability reagent (Promega) at 0 h and 72 h according to the manufacturer’s protocol. Each data point represents quadruplicates and experiments were repeated at least twice. IC50 values were calculated, using the GR calculator at www.grcalculator.org. Proliferation assays with lenalidomide were performed in triplicate and performed twice, with a concentration of 1 μM vs DMSO, cells were stained with PI, counted and medium was replaced every 48 h. Statistical significance was assessed by two way ANOVA and Bonferroni post-test. Detection of apoptosis was performed by Annexin V staining using the biolegend® FITC Annexin V Apoptosis Detection Kit with 7-AAD. Cell cycle analysis were performed with Hoechst33342 from SigmaAldrich® according to the manufacturer’s protocol.

### shRNA toxicity assay

Tet-pLKO-puro was a gift from Dmitri Wiederschain (Addgene plasmid # 21915). We exchanged the sequence of IRES-PuroR in the plasmid with that of P2A-EGFP using gBlocks® from Integrated DNA Technologies. Each shRNA was cloned into the Tet-pLKO-EGFP vector according to the protocol provided on the webpage (https://media.addgene.org/data/plasmids/21/21915/21915-attachment_Jws3xzJOO5Cu.pdf). We induced each plasmid into the target cells by lentivirus. psPAX2 and pCMV-VSV-G were used for lentiviral transduction. psPAX2 and pCMV-VSV-G were gifts from Didier Trono and Bob Weinberg (Addgene plasmids # 12260 and # 8454, respectively). Four days after the transduction, shRNA was induced by administration of doxycycline (final 1 μg permL, Clontech, Cat no. 631311). Fractions of GFP were measured over time by flow cytometry (BD FACSCanto II HTS, BD Biosciences).

The sequences used for shRNAs are:

shCtrl; CTCTCAACCCTTTAAATCTGA

shIRF4-1; CCGCCATTCCTCTATTCAAGA

shIRF4-2; GTGCCATTTCTCAGGGAAGTA

shMDM2; GATTCCAGAGAGTCATGTGTT (TRCN0000003377)

shMDM4; CACCTAGAAGTAATGGCTCAA (TRCN0000003857)

### Fluorescence in situ hybridization

A custom two-color dual-fusion FISH probe designed for the detection of inversion or translocation between *TBL1XR1* at 3q26.32 and *TP63* at 3q28 was designed using BAC probes RP11-1148M8 and RP11-1065H24 (surrounding *TBL1XR1* and labeled in green) and RP11-24F1 and RP11-53D15 (surrounding *TP63* and labeled in red). The probe set was hybridized to SMZ-1, DL40, MTA, OCI-Ly12, and OCI-Ly13.2. For the HH cell line, a custom two-color breakapart FISH probe was used to detect translocation between *TP63* at 3q28 and any other partner gene using BAC probes surrounding *TP63*: RP11-24F1 labeled in green and RP11-53D15 labeled in red. To detect *TCL1* rearrangements, we utilized the Cytocell TCL1 Breakapart probe LPH 046.

### Histology and immunohistochemistry

PDX specimens were fixed in 10% neutral-buffered formalin for 48 h and stored in 70% ethanol for up to 2 weeks before paraffin embedding. Immunohistochemical studies were performed on 4 μm paraffin sections using the following antibodies: CD3 (Yurogen, #916-MSM8-P0), CD4 (Abcam, #ab133616), p21 (CST, #2947), CD30 (Yurogen, #943-MSM1-IHC7), CD10 (Angio-Proteomie, #hAP-30576), PD1 (Yurogen, #5133-MSM1-P0), CD20 (Yurogen, #931-MSM2-CSO), CD7 (Abcam, #ab109296), CD8 (Abcam, #ab93278), CD57 (Abcam, #ab187274), pJAK2 (Abcam, #ab32191), and pSTAT5 (Abcam, #ab208043). In situ hybridization for Epstein–Barr virus encoded RNA (EBER) was performed using the Pan Path EBER Kit (#A500K.9901).

### RT-PCR

SuperScript III Reverse Transcriptase (Thermo Fisher Scientific) was used to prepare cDNA per manufacturer’s protocol. Primers for TBL1XR1-TP63 were F, ttgcaagagtcaggattttctca, and R, gaggagccgttctgaatctg. Primers for FOXK2-TP63 were F, tcttcagggtacaaggtggg, and R, gaggagccgttctgaatctg. Primers for ACTB were F, gctcgtcgtcgacaacggctc and R, caaacatgatctgggtcatcttctc.

### Western blot analysis

Sample preparation of whole cell lysates, SDS-PAGE, membrane transfer and blotting were performed according to standard protocols. Antibodies to p21 (#2947), PTPN2 (TCPTP) (#58935), Jak2 (D2E12) (#3230), Phospho-Jak2 (Tyr1007/1008) (#3771), Stat3 (D1B2J) (#30835), phospho-Stat3 (Tyr705) (D3A7) (#9145), Stat5 (D3N2B) (#25656), phospho-Stat5 (Tyr694) (D47E7) (#4322), MDM2 (D1V2Z) (#86934), α-Tubulin (#2144) and GAPDH (#5174) were purchased from Cell Signaling Technologies. Antibody to TP53 (sc-47698) and MDMX (G-10) (sc-74467) were purchased from Santa Cruz Biotechnology. Antibodies were used at 1:1000 dilution (Cell Signaling Technologies) or at 1:500 dilution (Santa Cruz Biotechnology).

Uncropped western blots from the main figures are provided in Supplementary Figure 8.

### Whole exome and targeted sequencing

Whole exome sequencing (WES) bam files for four cell lines (HH, Karpas 299, SR-786 and SU-DHL-1) were downloaded from the Sanger Institute (http://cancer.sanger.ac.uk/cell_lines, EGA accession number: EGAD00001001039) GDSC dataset^62^. For two additional cell lines (SUP-M2 and MJ), CCLE WES bam files were used (https://portal.gdc.cancer.gov/legacy-archive). For the remaining cell lines, Whole exome sequencing was performed by the Cancer Cell Line Encyclopedia Platform at the Broad Institute according to standard protocols of the Cancer Cell Line Encyclopedia. Briefly, DNA was extracted using the QIAGEN® QIAamp DNA Mini Kit according to the manufacturer’s protocol. Prior to library preparation, DNA was fragmented (Covaris sonication) to 250 bp and further purified using Agentcourt AMPure XP beads. Size-selected DNA was then ligated to specific adapters during library preparation (Kapa Library Prep, Kapa Biosystems). Each library was made with sample-specific barcodes. Libraries were pooled and sequenced over a HiSeq3000.

All the raw sequencing data was processed using CCLE variant calling pipeline. Mutation analysis for single nucleotide variants (SNVs) was performed using MuTect v1.1.6 in single sample mode with default parameters. Short indels were detected using Indelocator (http://archive.broadinstitute.org/cancer/cga/indelocator) in single sample mode with default parameters. To ensure high quality variant calls, we required minimum coverage of 4 reads with minimum two reads supporting the alternate allele. Moreover, variants with low allelic fraction (AF < 0.1) and variants outside protein coding region were excluded. To remove germline-like variants, any variant with allelic frequency greater than 1E-5 in Exome Aggregation Consortium (ExAC) project^63^ were excluded with the exception of any cancer recurrent variant defined by minimum TCGA frequency of 3 or COSMIC frequency of 10.

For targeted exon capture and next-generation sequencing of all coding exons of 265 genes previously implicated in PTCL (Supplementary Table 4), genomic DNA was extracted from banked xenografted tumor cells harvested from ≥ P1 mice following immunomagnetic depletion of murine cells (EasySep Mouse/Human Chimera Isolation Kit, #19849, StemCell Technologies, Vancouver, BC, Canada). DNA underwent customized hybrid-capture target enrichment (SureSelect, Agilent, Santa Clara, CA, USA) and Illumina sequencing. Genomic DNA from the splenocytes of a normal NSG mouse was sequenced in order to enhance species-specific filtering of human reads. Known germline polymorphisms from the Exome Sequencing Project and the dbSNP (build 142) databases were excluded. We applied the MutSigCV algorithm to identify genes that were altered more often than expected by chance given the background mutation rate. We only considered variants predicted to be non-silent (i.e., missense, nonsense, translational start site, or splice site alterations, or in-frame or frame-shift insertions/deletions) and with variant allele frequencies of 5% or more. For every alteration meeting these criteria, sequencing reads were individually visualized to evaluate mapping quality as well as the phase and spatial distribution of alterations within reads. Mutations were then called by cross-referencing candidate variants to ClinVar, COSMIC, and a review of published literature.

To validate mutations, cell lines and PDX tumors were sequenced using the Rapid Lymphoma Panel, an amplicon-based resequencing platform levying a custom panel of oligonucleotide probes which target a bevy of genes (1420 positive and negative strand probes for 98 gene targets, Supplementary Table 5) frequently implicated in lymphoma. Particular attention was paid to frameshifts, inframe indels, and nonsynonymous mutations. Paired-end sequencing of the ~300 bp amplicons is performed using an Illumina MiSeq, and Variant Call Format (VCF) files containing hits were generated using GRCh7/hg19 as the reference genome. Analysis of VCF files was performed using a combination of manual curation and as well as GeMSTONE, a cloud-based tool developed for variant filtering and annotation.

### Whole transcriptome sequencing

Analysis of RNASeq data was implemented as a Snakemake^64^ workflow. The complete workflow documenting all utilized parameters and tools so as to permit reproducibility is available at https://bitbucket.org/cfce/viper. Paired end RNASeq samples were mapped to the human genome reference assembly (hg19) with STAR 2.4.2a. Transcript expressions were estimated with Cufflinks 2.2.1 without transcript assembly^65^. Gene expressions were calculated as sums of transcript FPKM values. Genes with transcripts smaller than 300 bp were ignored. All expression values were log2 transformed. For gene fusion detection, we use STAR-Fusion (https://github.com/STAR-Fusion/STAR-Fusion^66^). It is a method that accurately identifies fusion transcripts from RNA-Seq data and outputs all supporting data discovered during alignment. We used a cutoff of 5 reads (either spanning or crossing the fusion) to call the presence of a translocation.

For the Sample–Sample Correlation heatmap (Supplementary Figure 3) we used the expression matrix from Cufflinks^67^. All snoRNA and miRNA genes were first filtered out as were all genes that were only significantly expressed in 3 or fewer samples based on a FPKM threshold of 5. A custom R script then calculates the Pearson correlation between all of the samples on a pairwise basis and generates the heatmap.

For GSEA, differential expression between experimental criteria was determined using raw counts and normalization procedures within the DESeq2 R package, using a negative binomial distribution. Genes were ordered by the Wald statistics and these ordered lists were used in GSEA (Broad Institute). The false discovery rate (FDR—Benjamini and Hochberg) method was used to adjust for multiple comparisons.

To permit analysis of the aggregated cell line and PDX data, we first performed quantile normalization to adjust for library depth and platform-specific differences. Principal components analysis revealed that the primary source of post-normalization variation derived from batch effects. These batch effects within PDX and Cell Line were successfully removed using the ComBat approach from SVA 3.18.00^68^.

### Archer fusion panel

An Anchored Multiplex PCR (AMP) assay was used for targeted fusion transcript detection using next generation sequencing (NGS). Briefly, total nucleic acid was isolated from cell pellets, blood, bone marrow aspirate or smears. The total nucleic acid was reverse transcribed with random hexamers, followed by second strand synthesis to create double-stranded complementary DNA (cDNA). The double-stranded cDNA was end-repaired, adenylated, and ligated with a half-functional adapter. Two hemi-nested PCR reactions using the ArcherDx Heme Fusion kit primers were performed to create a fully functional sequencing library that targets specific genes (exons, Supplementary Table 6). Illumina NextSeq 2 × 150 base paired-end sequencing results were aligned to the hg19 human genome reference using bwa-mem^69^. A laboratory-developed algorithm was used for fusion transcript detection and annotation. The integrity of the input nucleic acid and the technical performance of the assay were assessed with a qualitative reverse transcription qPCR assay and assessing the DNA/RNA content in the sequencing results. Although this assay may detect several potential fusion variants, only the most prevalent one is reported. The assay is validated for samples showing 6% or higher tumor cellularity.

### CRISPR-Cas9-screen

Genome-scale CRISPR-Cas9 loss-of-function screening was performed on 391 cancer cell lines including 8 T-cell lymphoma cell lines using the Avana library^70, 71^. Briefly, cancer cell lines were transduced with Cas9 using a lentiviral system^71^. Cell lines that met criteria, including acceptable Cas9 activity measuring ability to knock out transduced GFP, appropriate growth properties and other parameters, were then screened with the Avana library. The Avana library^72^ designed at the Broad Institute contains >70,000 sgRNAs (single guide RNAs) and an average of 4 guides per gene with approximately 1000 guides that do not target any location in the reference genome as negative controls. A pool of guides was transduced into a population of cells. The cells were cultured for approximately 21 days in vitro, and at the end of the assay, barcodes for each guide were sequenced for each cell line in replicate. Reads per kilobase were calculated for each replicate and then the log_2_ fold change compared to the initial plasmid pool was calculated for each guide. Samples with poor replicate reproducibility (<0.7 Pearson coefficient), total read counts less than 15 million, or those that failed to match the fingerprint of parent lines were removed from the analysis. Additionally, guides that have low representation in the initial plasmid pool and those with suspected off-target activity were removed from analysis. Dependency scores were generated for each gene in each cell line using the CERES algorithm^70^. Briefly, CERES estimates gene dependency levels from CRISPR-Cas9 essentiality screens while accounting for the copy-number-specific effect, as well as variable sgRNA activity. The gene dependency scores generated by CERES are absolute dependency scores with each cell line scaled to have an average dependency score of 0 for negative control sgRNAs and an average dependency score of −1 for a set of positive control core essential genes as defined by Hart et al.

To calculate the probability and false-discovery rate (FDR) that a gene dependency score represents a true dependency in a given cell line, we fit a two-component mixture model in each cell line. The two components were 1 the empirically determined distribution of true dependent scores, identified using the pan-essential gene scores in that cell line, and 2 the empirically determined distribution of true non-dependent scores, identified as genes that were not expressed in that line. We defined as pan-essential genes 1871 genes whose dependency scores falls in the bottom 24% of gene scores in at least 90% of the cell lines. The probability of dependency for each gene score is the probability that it was generated from the distribution of true dependent gene scores. To correct for noise in the tails of the distributions, all gene scores below −1.5 were assigned probability 1 of being dependencies and all gene scores above 0.25 were assigned probability 0. A Gaussian smoothing kernel with width 0.15 was applied to the final probability scores to further reduce noise.

In order to identify outlier dependencies in T-cell lymphoma lines compared to the other cancer cell lines screened, *Z*-scores were calculated for each gene across all cancer cell lines using the median dependency score for each gene and the median average deviation. Genes with *Z*-scores <−4 in at least 2 T-cell lymphoma lines with an FDR < 0.05 in at least 1 T-cell lymphoma line with expression of the gene >1 RPKM were considered candidate T-cell lymphoma dependencies.

### In vitro competition assay

KI-JK and SMZ-1 were transduced with Cas9 as described in “CRISPR-Cas9-screen”. Each sgRNA targeting PTPN2 (PTPN2 #1, PTPN2 #2) was cloned into the pLKO5-U6-EF1a-RFP657 and the Control sgRNA was cloned into pLKO5-U6-EF1a-GFP, which were gifts from Benjamin Ebert (Addgene plasmid # 57822 and # 57824). We introduced each sgRNA into the target cells with lentiviral transduction. Three days after infection, Cas9-target sgRNA-transfected cells with RFP657 and Cas9-control sgRNA-transfected cells with GFP were mixed. Culture media was changed every 3–4 days and the fractions of RFP657- and GFP-expressing cells were quantified on day 9 by flow cytometry (BD FACSCanto II).

The sequences used for sgRNAs were:

PTPN2 #1:CCATGACTATCCTCATAGAG

PTPN2 #2:CCGCGACTCACCAAGTACAG

Control:GCACTACCAGAGCTAACTCA

### In vivo studies

Viably frozen patient-derived xenograft cells were thawed and washed in 1 × PBS before tail-vein injection at 0.7–1.7 × 10^6^ cells per mouse. For subcutaneous models, tumor fragments were implanted in the right flank under isoflurane anesthesia. Tumor burden was monitored periodically based on engraftment kinetics of each model by flow cytometry of peripheral blood, analysis of target organ involvement from sentinel animals for non-circulating models, or tumor size by caliper measurement. Blood was processed with Red Blood Cell Lysis Buffer (Qiagen) before staining with antibodies against human CD45 (Pacific Blue V450-conjugated, BD Bioscience 560367) and human CD2 (APC-conjugated, BioLegend 300213) or human CD56 (APC-conjugated, BD Bioscience 555518) in 1 × PBS with 1 mM EDTA plus 1% fetal bovine serum. All antibodies were used at a dilution of 1:20. Flow cytometry data was analyzed with FlowJo.

Upon engraftment, mice were randomized to vehicle or treatment arms. Ruxolitinib was given at 90 mg per kg in PBS + 0.1% Tween 20, orally by gavage twice per day; romidepsin at 1 mg per kg diluted in PBS, given intraperitoneally on days 1, 4, and 7; ALRN-6924 at 20 mg per kg diluted in 20 mM sodium phosphate, 240 mM trehalose and 0.03% (w per v) polysorbate 20, pH 7.5 given intravenously on days 1, 4, and 7. The vehicle for romidepsin and ALRN-6924 was PBS intraperitoneally and ALRN6924 vehicle intravenously on the same schedule as the drugs.

All drugs were dosed in 10 mL per kg volume based on body weight. Mice were humanely sacrificed 16 h after the final dose; blood and target organs were collected for complete blood count, immunohistochemistry, and flow cytometry to determine tumor burden using the same antibodies as above. All animal work was performed in Nod.Cg-Prkdc^scid^IL2rg^tm1Wjl^/SzJ (NSG) mice purchased from Jackson Laboratories and handled according to Dana-Farber Cancer Institute’s Institutional Animal Care and Use Committee approved protocol #13–034.

### Contribution of human participants

For PDX generation, de-identified patient samples were obtained with informed consent and xenografted under Dana-Farber/Harvard Cancer Center Institutional Review Board (IRB)-approved protocol #13–351. The patient reported in Fig. 3 participated in the phase 1/2a trial of ALRN-6924 (NCT02264613). Informed consent was obtained according to the study protocol and was approved by the University of Alabama-Birmingham Cancer Center Institutional Review Board.

### Data availability

All primary data from RNAseq, exome sequencing, and CRISPR screening are available in Supplementary Data 7–10. Raw data files are available at https://portal.gdc.cancer.gov/legacy-archive/search/f and have been deposited in NCBI's Gene Expression Omnibus and are accessible through GEO Series accession number GSE114085^73^. Mutation and RNASeq data for PDXs are also available at www.PRoXe.org. All other remaining data are available within the article and supplementary files, or available from the authors upon request.

## Electronic supplementary material

Supplementary Information

Peer Review File

Description of Additional Supplementary Files

Supplementary Data 1

Supplementary Data 2

Supplementary Data 3

Supplementary Data 4

Supplementary Data 5

Supplementary Data 6

Supplementary Data 7

Supplementary Data 8

Supplementary Data 9

Supplementary Data 10
