# Supplementary material for: Targetable vulnerabilities in T- and NK-cell lymphomas identified through preclinical models
Source: Nat Commun. 2018 May 22;9:2024. doi: 10.1038/s41467-018-04356-9 (PMC5964252; doi:10.1038/s41467-018-04356-9)
Supplement: Supplementary file 1 — Supplementary Information [file 41467_2018_4356_MOESM1_ESM.pdf]

Supplementary Figure 1

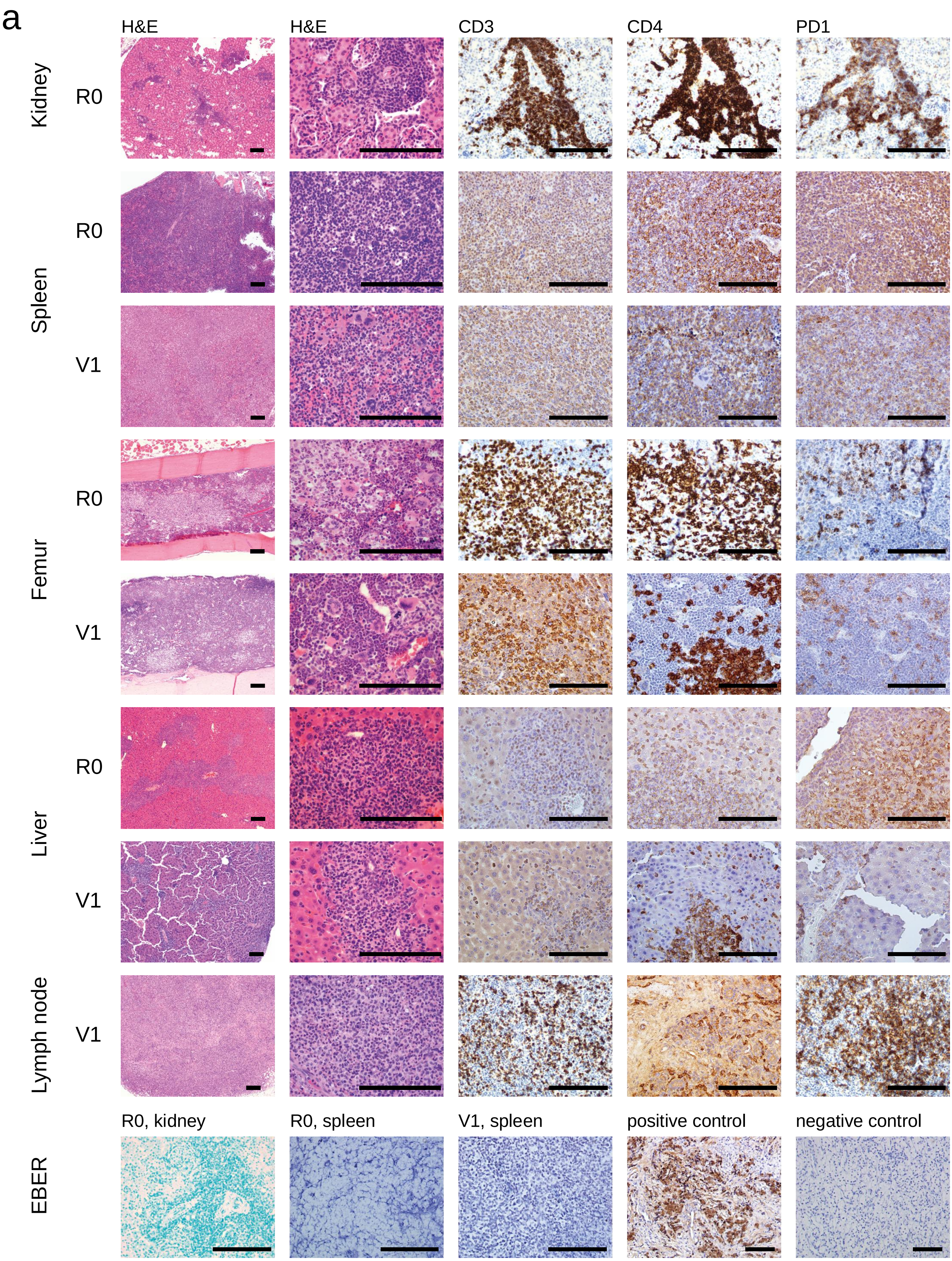

b

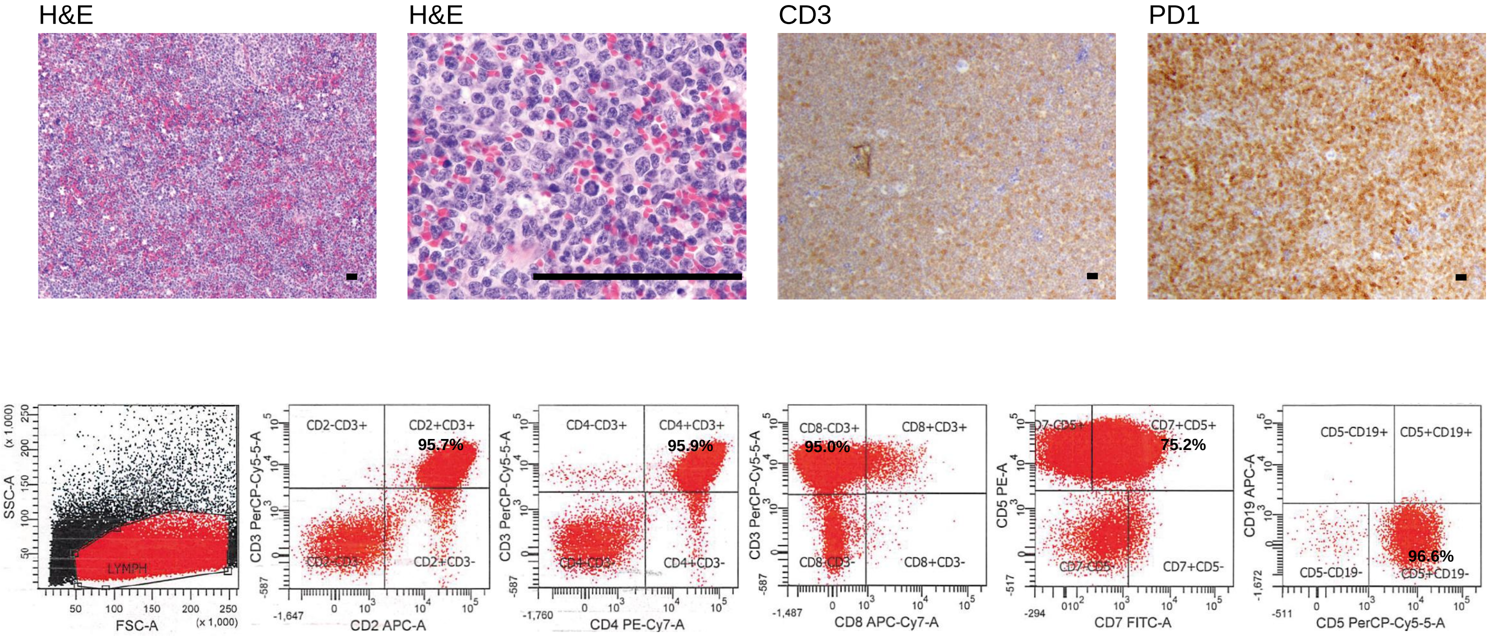

H&E
H&E
CD3
PD1
SSC-A
FSC-A
LYMPH
CD3 PerCP-Cy5-5-A
CD2 APC-A
CD2-CD3+
CD2+CD3+
95.7%
CD2+CD3-
CD4 PE-Cy7-A
CD4-CD3+
CD4+CD3+
95.9%
CD4+CD3-
CD8 APC-Cy7-A
CD8-CD3+
CD8+CD3+
95.0%
CD8-CD3-
CD8+CD3-
CD5 PE-A
CD7 FITC-A
CD7+CD5+
75.2%
CD7+CD5-
CD19 APC-A
CD5 PerCP-Cy5-5-A
CD5-CD19+
CD5+CD19+
CD5-CD19-
96.6%
CD5+CD19-

c

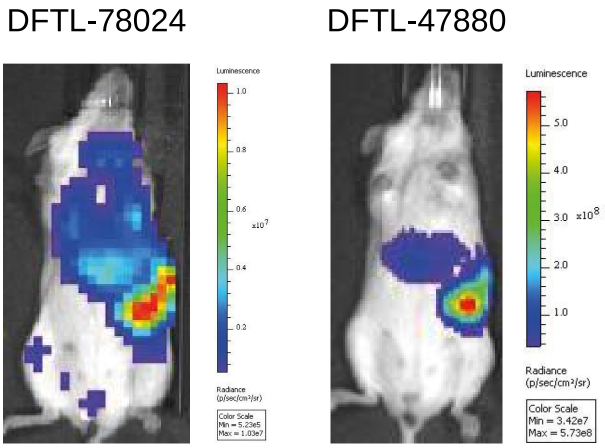

DFTL-78024
DFTL-47880
Luminescence
Radiance (p/sec/cm²/sr)
Color Scale Min = 5.23e5 Max = 1.03e7
Color Scale Min = 3.42e7 Max = 5.73e8

d

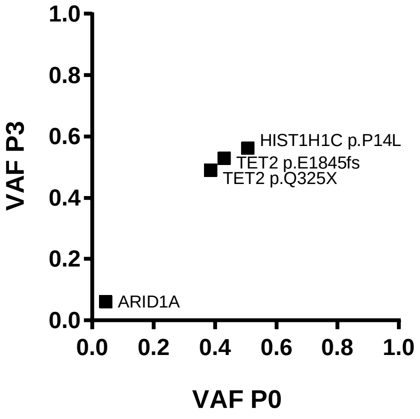

VAF P3
VAF P0
HIST1H1C p.P14L
TET2 p.E1845fs
TET2 p.Q325X
ARID1A

e

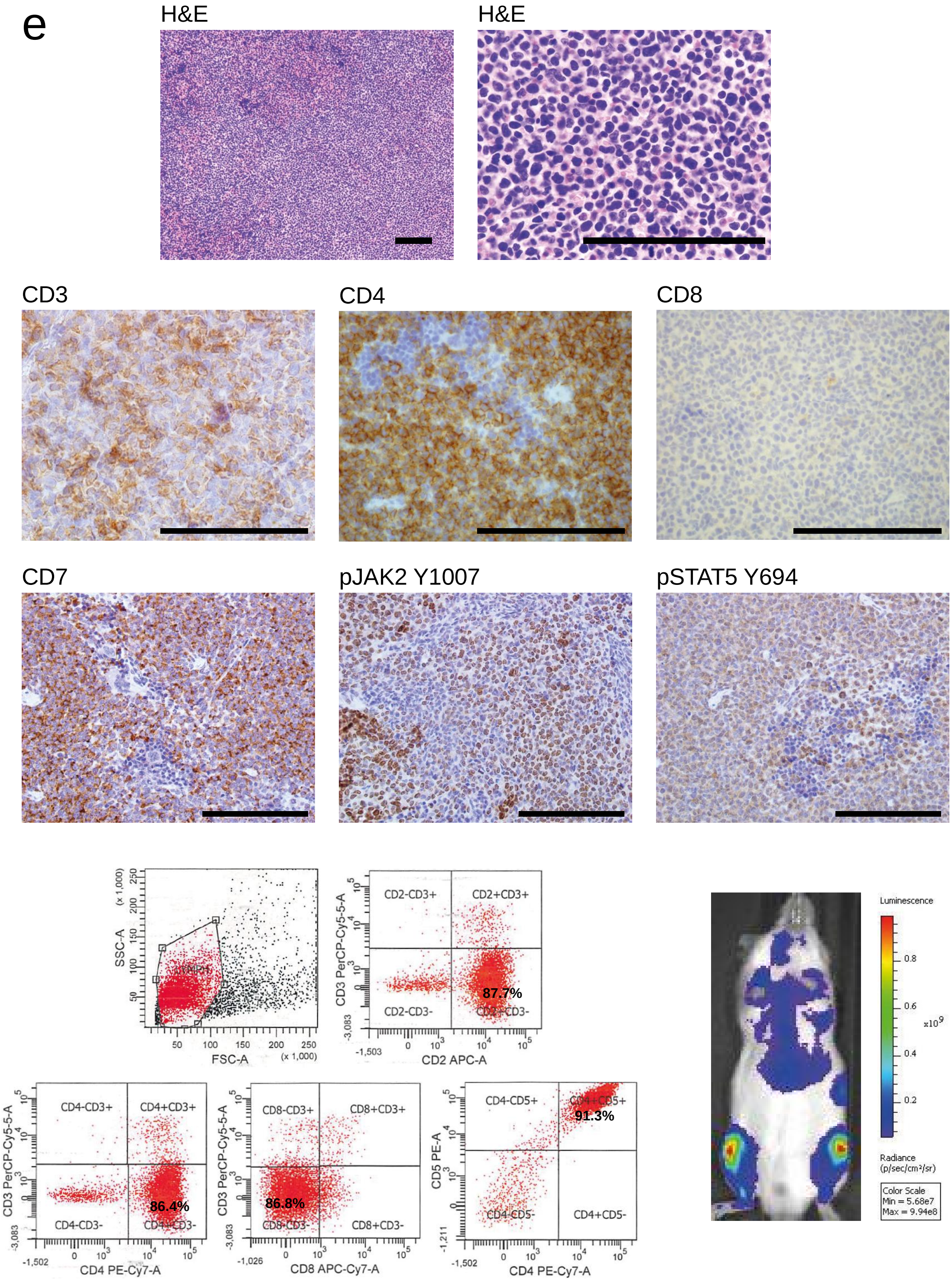
H&E
H&E
CD3
CD4
CD8
CD7
pJAK2 Y1007
pSTAT5 Y694
SSC-A
FSC-A
(x 1,000)
CD2-CD3+
CD2+CD3+
CD2-CD3-
87.7%
CD3 PerCP-Cy5-5-A
CD2 APC-A
CD4-CD3+
CD4+CD3+
CD4-CD3-
CD4+CD3-
86.4%
CD4 PE-Cy7-A
CD8-CD3+
CD8+CD3+
CD8-CD3-
CD8+CD3-
86.8%
CD8 APC-Cy7-A
CD4-CD5+
CD4+CD5+
91.3%
CD4-CD5-
CD4+CD5-
CD5 PE-A
Luminescence
Radiance
(p/sec/cm²/sr)
Color Scale
Min = 5.68e7
Max = 9.94e8

f

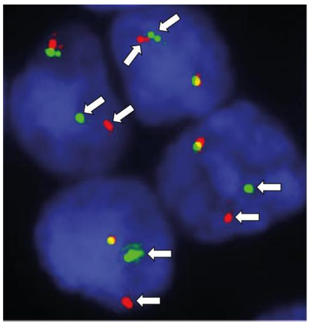

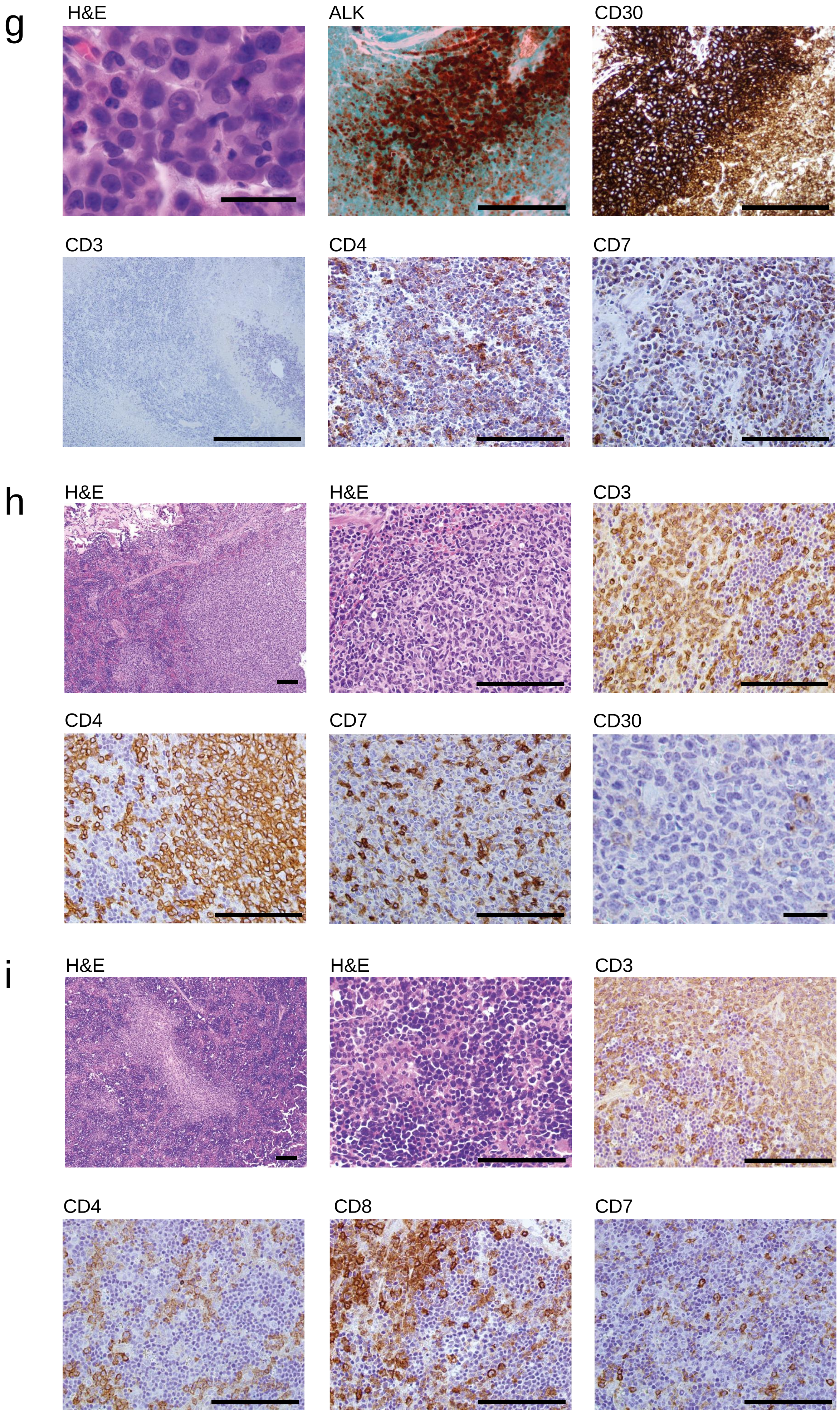
g
H&E
ALK
CD30
CD3
CD4
CD7
h
H&E
H&E
CD3
CD4
CD7
CD30
i
H&E
H&E
CD3
CD4
CD8
CD7

j

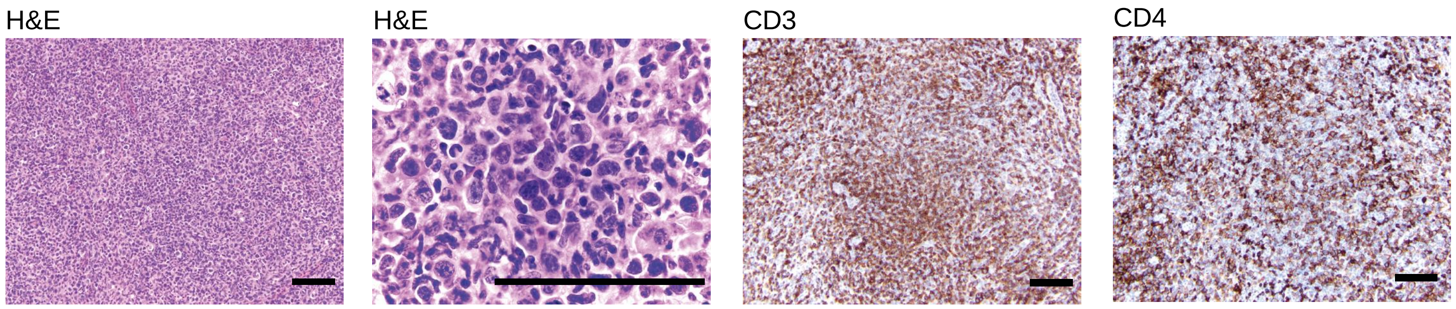
H&E
H&E
CD3
CD4

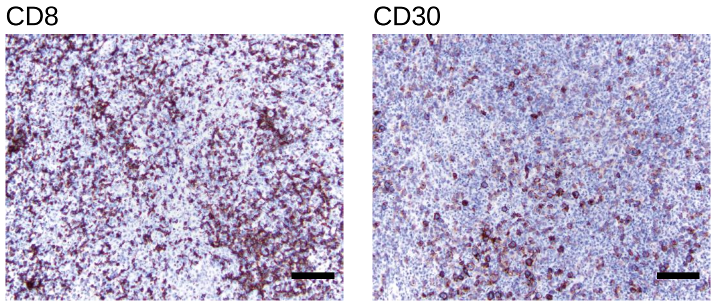
CD8
CD30

k

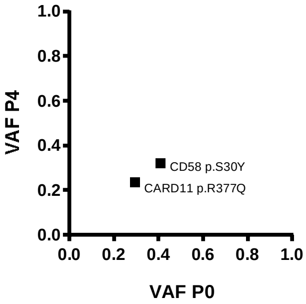
VAF P4
VAF P0
1.0
0.8
0.6
0.4
0.2
0.0
CD58 p.S30Y
CARD11 p.R377Q

l

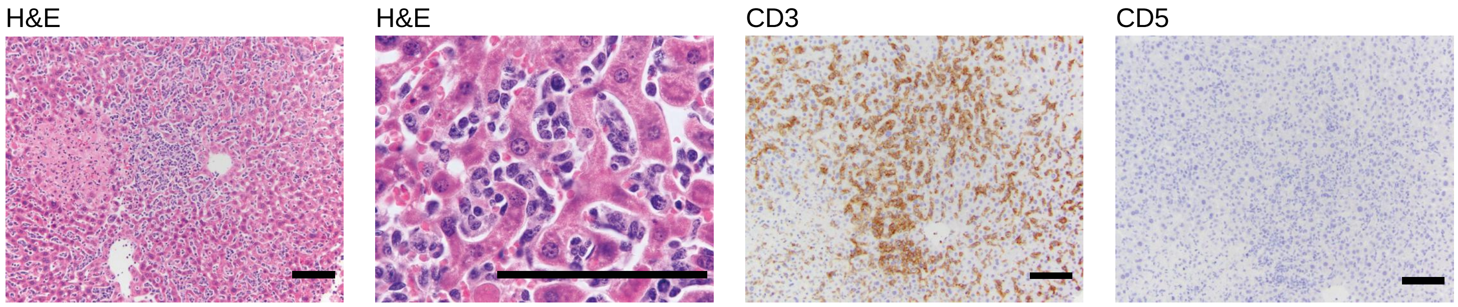
H&E
H&E
CD3
CD5

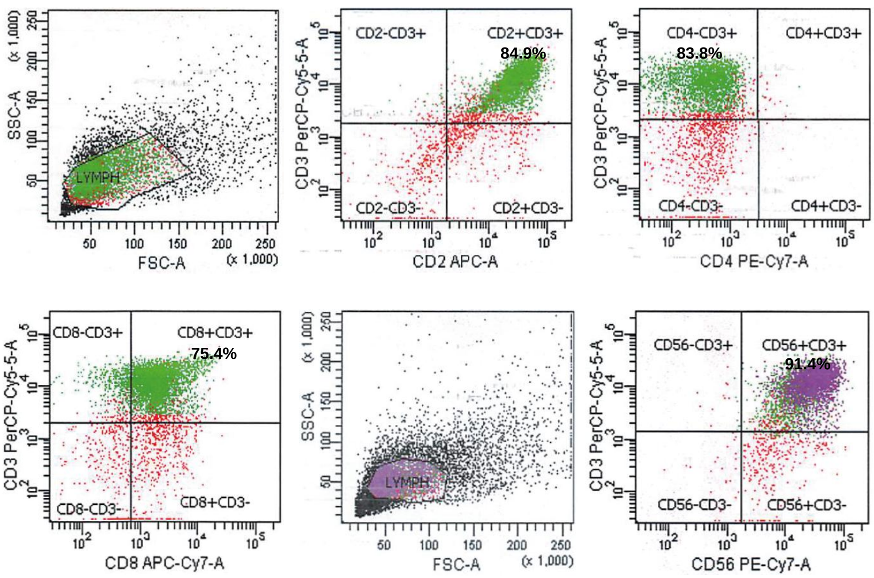
SSC-A
FSC-A
LYMPH
CD2-CD3+
CD2+CD3+
84.9%
CD2-CD3-
CD2+CD3-
CD2 APC-A
CD4-CD3+
CD4+CD3+
83.8%
CD4-CD3-
CD4+CD3-
CD4 PE-Cy7-A
CD8-CD3+
CD8+CD3+
75.4%
CD8-CD3-
CD8+CD3-
CD8 APC-Cy7-A
CD56-CD3+
CD56+CD3+
91.4%
CD56-CD3-
CD56+CD3-
CD56 PE-Cy7-A
CD3 PerCP-Cy5-5-A

m

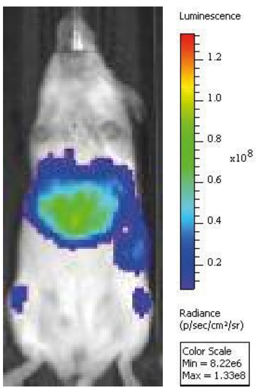
Luminescence
Radiance (p/sec/cm²/sr)
Color Scale
Min = 8.22e6
Max = 1.33e8

Supplementary Figure 1: Phenotypic characterization of PDX models

a) AITL xenograft DFTL-78024: The patient's lymphoma showed expression of CD2, CD3, CD4, CD5, TCRα/β, CD56 and PD-1, and was negative for CD7, CD8, Epstein Barr Virus-encoding RNAs (EBER) and additional NK-cell markers (CD16, CD57, CD94). A lymph node biopsy at progression post chemotherapy was implanted in the renal capsule. The tumor engrafted in the adjacent kidney, the spleen, bone marrow and liver. Subsequent passages engrafted in the spleen, liver, bone marrow and lymph nodes upon intravenous injection and maintained the immunophenotype of the originating lymphoma.
b) AITL xenograft DFTL-47880: This model is derived from the peripheral blood from a patient with AITL at relapse after chemotherapy. The immunophenotype matches the reported infiltration of an axillary lymph node by a CD4-positive T-cell population with co-expression of CD2, CD3, CD5, CD7 and PD-1.
c) Bioluminescence imaging (BLI) of luciferized AITL cells reveals engraftment of DFTL-78024 in the spleen, liver, lungs, cervical lymph nodes and the bone marrow. DFTL-47880 shows strong splenic and moderate liver infiltration.
d) Targeted DNA-sequencing reveals clonal stability of pathogenic TET2, ARID1A and HIST1H1C mutations in DFTL-78024. Notably, both TET2 mutations were previously identified in the originating patient's lymphoma.
e) T-PLL xenograft DFTL-28776: The morphology reveals a typical appearance for T-PLL with small to intermediate-sized cells exhibiting variably condensed chromatin. The patient's original immunophenotype (positive for CD5 (bright), CD2, CD4, CD7; dim to negative surface CD3 expression and dim CD8 expression) is maintained. By IHC, the malignant cells exhibit cytoplasmic CD3, CD4 and CD7 expression and strong JAK2 and STAT5 phosphorylation. BLI reveals infiltration of the spleen and widely spread bone marrow infiltration with luciferized DFTL-28776 cells.
f) A two-color break-apart FISH probe identifies a *TCL1* rearrangement in DFTL-28776.
g) ALK+ ALCL xenograft WCTL-81162: the malignant cells of this subcutaneous xenograft show a typical anaplastic large cell morphology. The tumor cells exhibit a strong expression of CD3, ALK and CD30, while both CD4 and CD7 are only partially expressed.
h) PTCL-NOS xenograft DFTL-35806: The reported immunophenotype of the patient is CD2+, CD3+, CD4weak, CD5subset and CD30+, and negative for CD5, CD7, CD10 and EBER: neg. Upon implantation of a tumor seed in the renal capsule, the spleen was infiltrated by a CD3 and CD4 positive population with partial expression of CD7 and weak CD30.
i) PTCL-NOS xenograft DFTL-82248: patient's immunophenotype is TCR-BF1, CD3, CD4 (subset), CD8 (small subset), CD2 (subset), CD5, CD7 and CD25 (small subset), scattered cells are positive for TCR-γ, CD20 highlights scattered interspersed small B-cells.
Upon subcutaneous implantation, a CD3 positive population with co-expression of CD4(subset), CD8 and CD7(subset) engrafted in the spleens of the mice.
j) CTCL-model DFTL-22685: The xenograft is derived from a patient with refractory systemic CTCL with CD30-positive large-cell transformation after gemcitabine and brentuximab. Upon tail vein injection of circulating tumor cells, large atypical cells infiltrated an inguinal lymph node. IHC reveals that the large tumor cells are positive for CD3, CD4 and CD30, and negative for CD8. There is a background of small T-cells, including both CD4-positive and CD8-positive

subsets, which lack expression of CD30.
k) Targeted DNA-sequencing reveals clonal stability of pathogenic CARD11 and CD58 mutations across 4 passages.
l) HS-TCL xenograft CBTL-81777: At diagnosis of this Hepatosplenic T-cell Lymphoma, The Boston Children's Hospital flow showed a malignant population "positive for CD45 and co-expresses CD2 (bright), surface CD3, CD56, CD7 (partial/small subset) and CD8(bright), but is negative for CD5, CD4, CD34, TdT, CD13, CD33 and negative for MPO". Upon tail vein injection, this xenograft engrafts in the liver and the spleen and it shows a characteristic sinusoidal growth pattern in the liver. Immunophenotyping by IHC and flow cytometry confirms the phenotype of the originating tumor.
m) BLI revealed infiltration of the liver, spleen and bone marrow with luciferized CBTL-81777 cells.
All scale bars indicate 0.1mm.

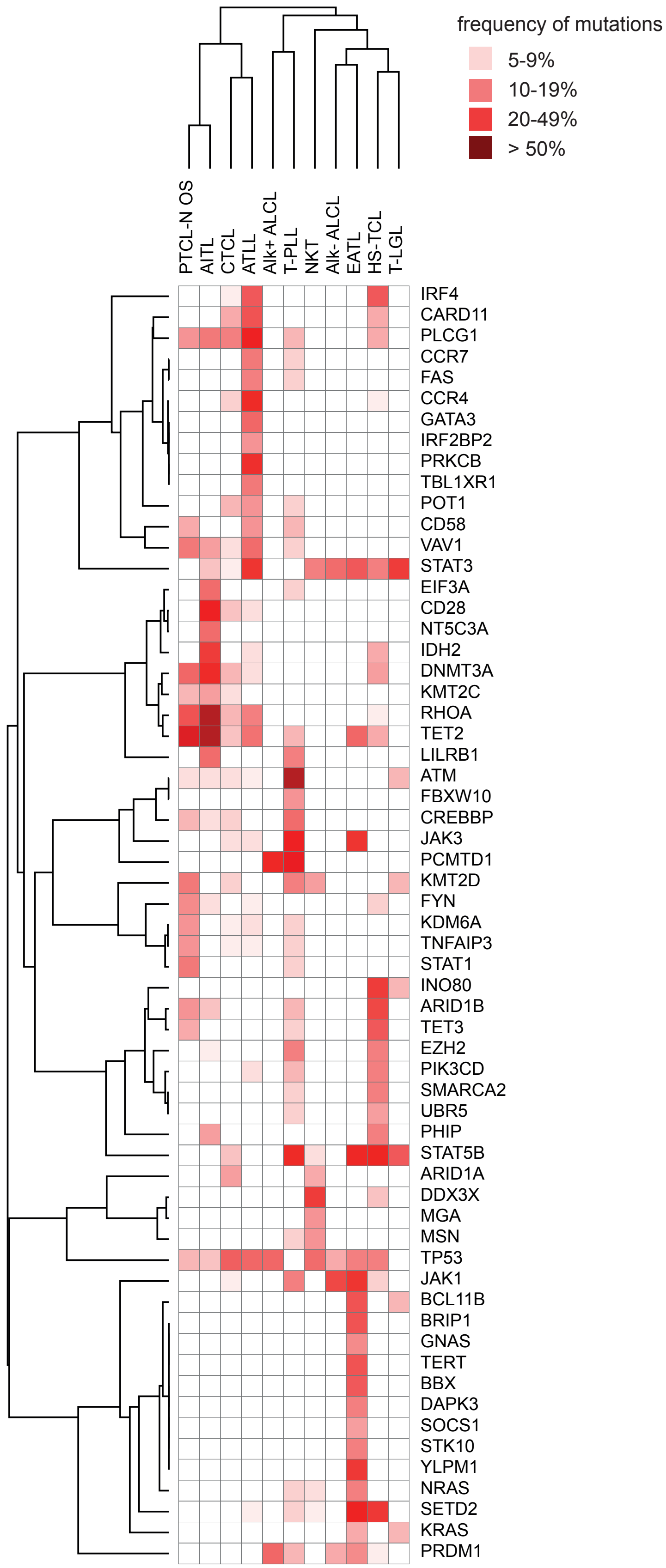

Supplementary Figure 2: Reported mutations in clinical TCL samples.

Unsupervised hierarchical clustering of reported recurrent mutations in clinical samples across subtypes of T- and NK-cell lymphomas, according to the literature referenced in Supplementary Table 3.

Supplementary Figure 3

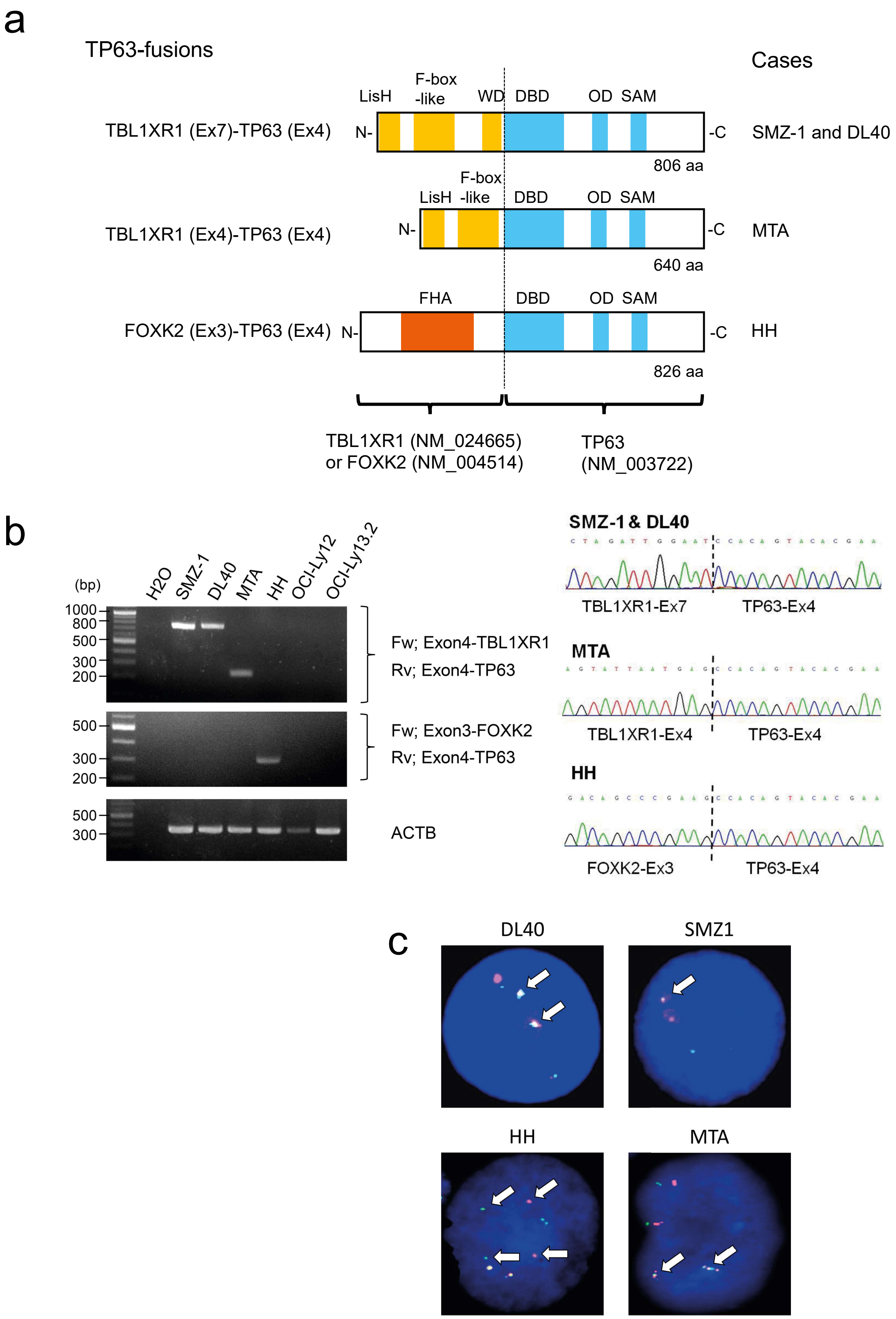

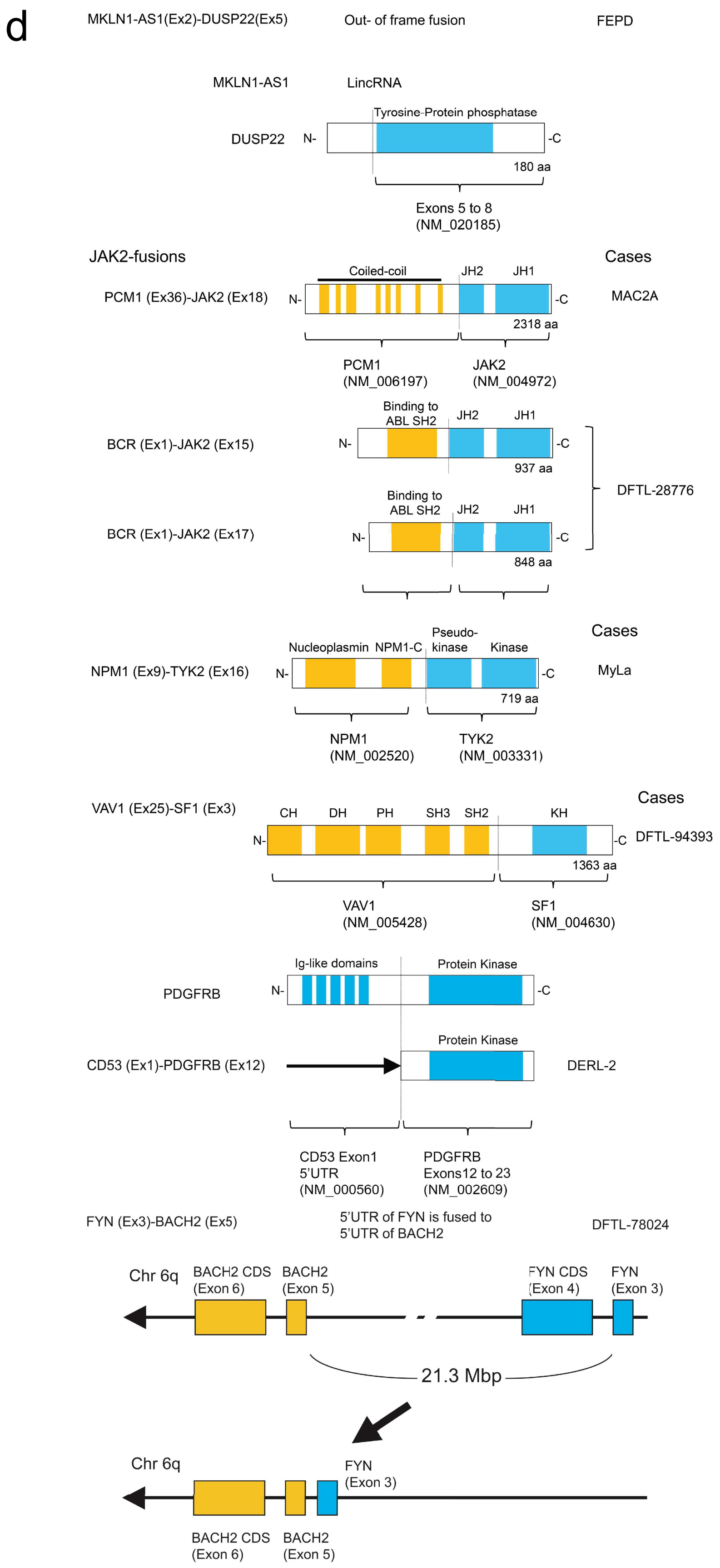

Supplementary Figure 3: Fusions

a) Graphical illustration of TP63 fusions.

b) RT-PCR and Sanger sequencing of TBL1XR1-TP63 and FOXK2-TP63 fusions. OCI-Ly12 and OCI-Ly13.2 served as negative controls.

c) FISH using a two-color dual-fusion FISH probe designed for the detection of inversion or translocation between TBL1XR1 at 3q26.32 and TP63 at 3q28. For HH, a custom two-color breakapart FISH probe was used. Each arrow indicates the fusion signal.

d) Graphical illustration of fusions involving DUSP22, JAK2, TYK2, VAV1, FYN and PDGFRB. In DFTL-94393 (AITL) the C-terminus of the SH3 domain of VAV1 is replaced by the C-terminus of SF1.

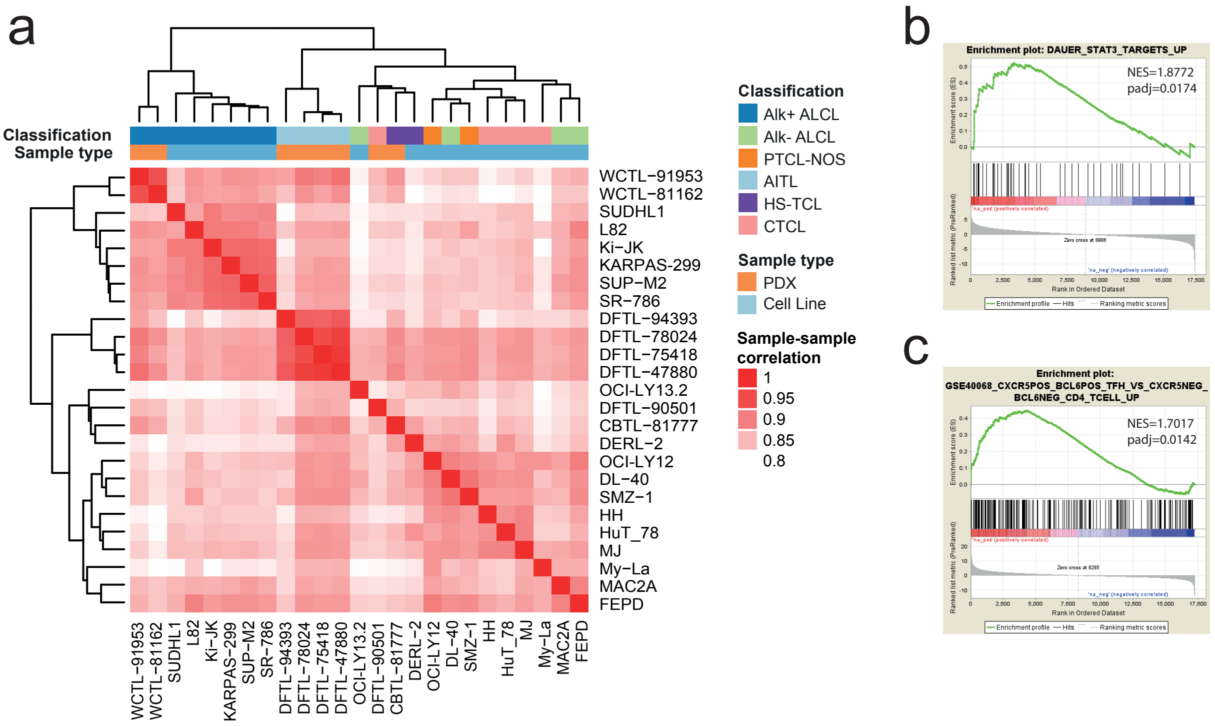

Supplementary Figure 4: Transcriptomic analysis

a) Sample-sample correlation plot clustered by the transcriptome of PDX models and cell lines.

b) GSEA comparing PDX models and cell lines of ALK-rearranged ALCL with PDX models and cell lines of the remaining subtypes reveals an enrichment of STAT3 target genes.

c) GSEA analysis comparing PDX models of AITL with PDX models of other PTCL subtypes identifies an enrichment of a follicular helper T-cell (T_{FH})-signature consistent with the AITL cell-of-origin.

Supplementary Figure 5

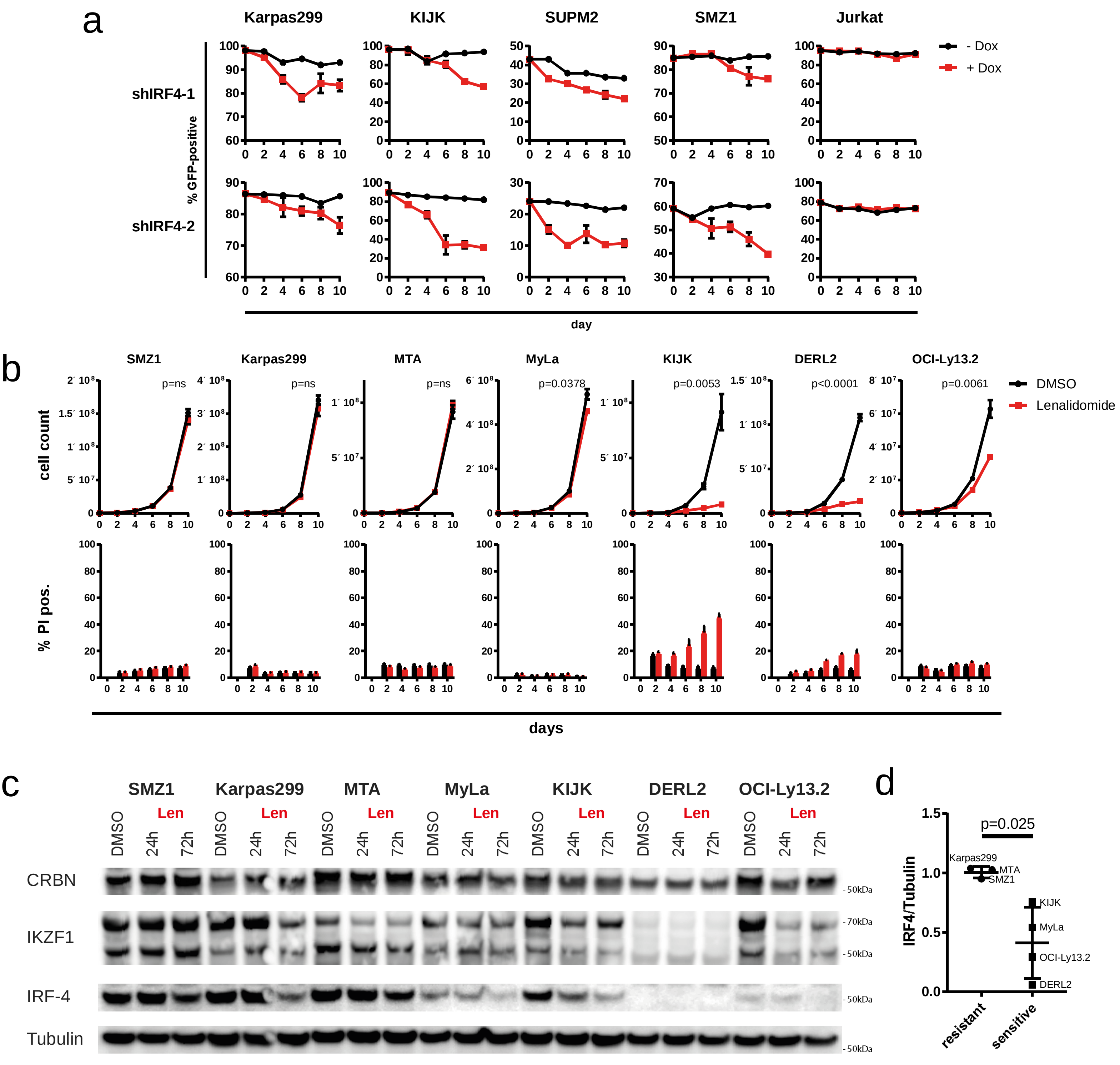

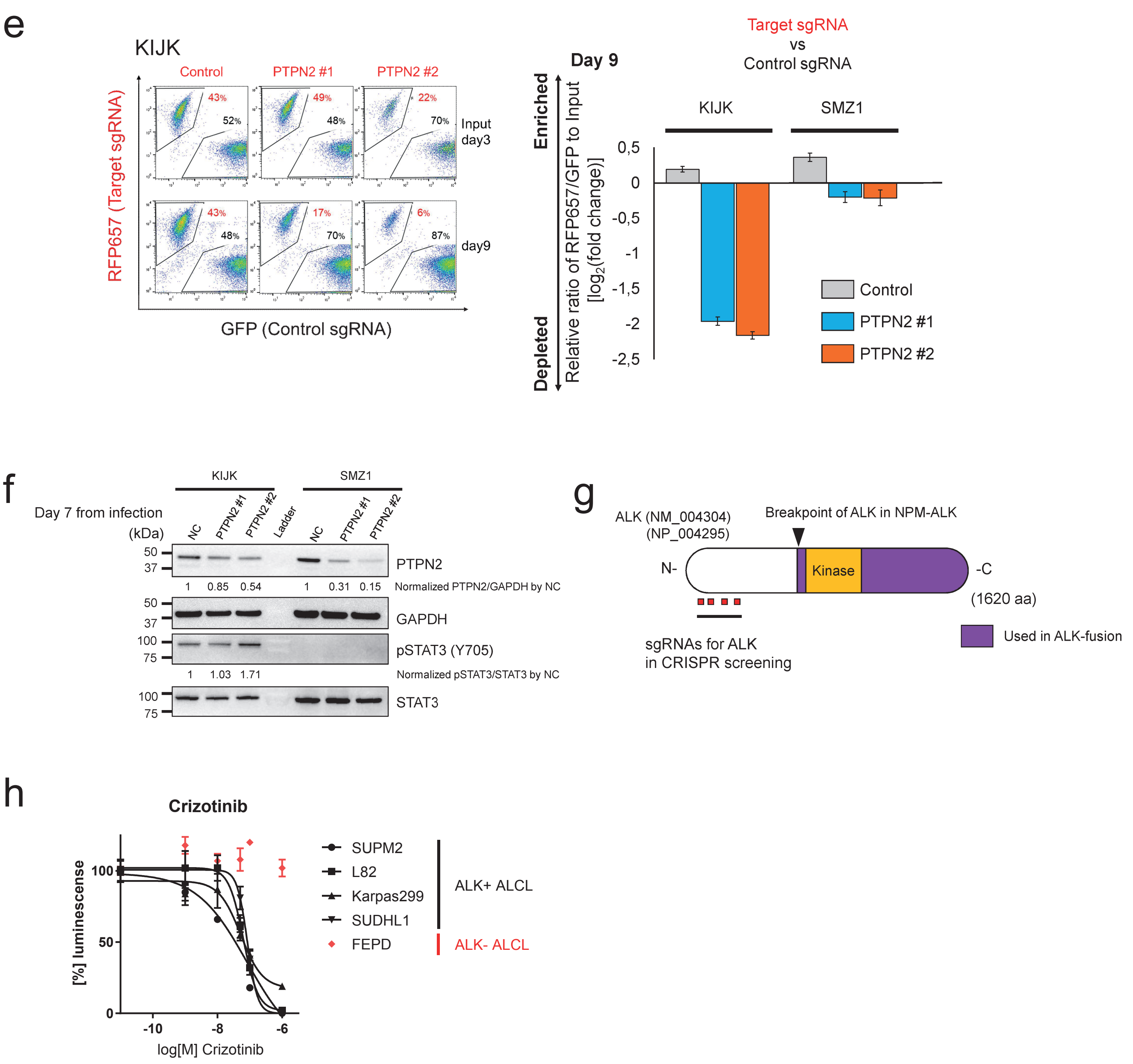

Supplementary Figure 5: Target validation of IRF4 and PTPN2

a) Knockdown validation of IRF4 using a GFP-expressing doxycycline-inducible shRNA system. Jurkat cells served as a negative control.

b) Cell proliferation and cytotoxicity in the presence of 1µM lenalidomide compared to DMSO. Comparisons are by 2-way ANOVA and Bonferroni correction for multiple comparisons.

c) Immunoblotting of CRBN, IKZF1 and IRF4 upon exposure to 1 µM lenalidomide for 24h and 72h.

d) Quantification of IRF4 protein abundance in cell lines resistant (SMZ1, Karpas299, MTA) or sensitive (MyLa, KIJK, DERL2, OCI-Ly13.2) to lenalidomide. Comparisons are by unpaired two-sided t-test.

e) Knockdown validation of PTPN2 utilizing a competition assay with a GFP-labeled control sgRNA and a RFP-labeled sgRNA targeting PTPN2. Based on the CRISPR-CAS9 screen results, SMZ1 served as a negative control.

f) Immunoblotting of PTPN2 in KIJK and SMZ1 after expression of CAS9 and sgRNA targeting PTPN2 or negative control (NC).

g) Illustration of ALK with its breakpoint in NPM-ALK fusions and localization of sgRNA targets.

h) In vitro activity of crizotinib in ALK+ ALCL cell lines SUPM2, L82, Karpas299 and SUDHL1 versus the ALK- ALCL cell line FEPD.

Representative data from two independent experiments performed in quadruplicates.

Data points in a), b), d) and e) are mean values of triplicates with error bars indicating standard error of the mean.

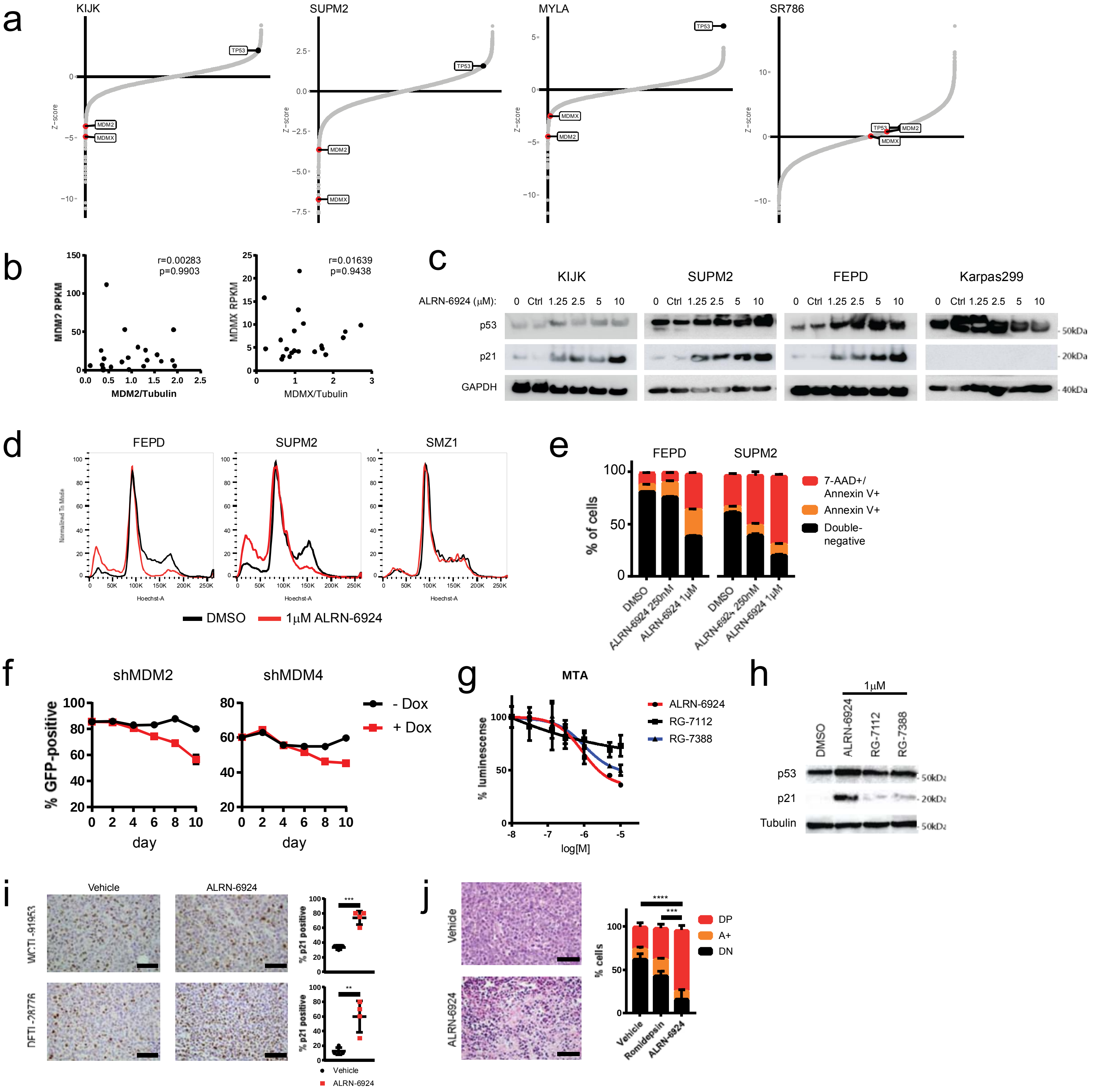

Supplementary Figure 6: MDM2, MDMX and ALRN-6924 in vitro and in vivo

a) Ranked dependency scores in individual cell lines with MDM2, MDMX and TP53 highlighted.

b) Lack of correlation between RNA expression (y axis) and protein expression normalized to tubulin (x axis) for MDM2 (left) and for MDMX (right). Each dot indicates a separate TCL line. c) Immunoblot of p53 and p21 in response to ALRN-6924 vs DMSO in TP53 wt (KIJK, SUPM2, FEPD) and TP53 mutated (Karpas299) cell lines after 24h. Control: ALRN-8753 at 10μM.

d) Cell cycle analysis by Hoechst33342 after 24h incubation with either DMSO or 1μM ALRN-6924, performed in triplicates

e) Annexin V/7-AAD staining after 24h incubation with either DMSO or ALRN-6924 at 250nM and 1μM. Representative data from two independent experiments performed in triplicates.

f) Knockdown validation of MDM2 and MDMX using a GFP-expressing tetracycline-inducible shRNA system in MTA cells. Performed in triplicates.

g) CellTiterGlo cytotoxicity assay after 72h incubation with ALRN-6924, RG-7112 and RG-7388 in MTA cells. Each datapoint was generated from a quadruplicate of samples.

h) Immunoblot of p21 and p53 in MTA cells treated with DMSO, or 1μM ALRN-6924, RG-7112 or RG-7388.

i) Representative IHC of p21 expression in vehicle-treated vs ALRN-6924-treated PDX models WCTL-91953 and DFTL-28776.

j) Representative H&E staining of vehicle-treated vs ALRN-6924-treated PDX model WCTL-81162. AnnexinV/7-AAD staining of WCTL-81162 treated with vehicle, romidepsin or ALRN-6924.

Data points in e), f), g), i), j) and k) are mean values with error bars indicating standard error of the mean. Scale bars in i), j) indicate 0.04mm.

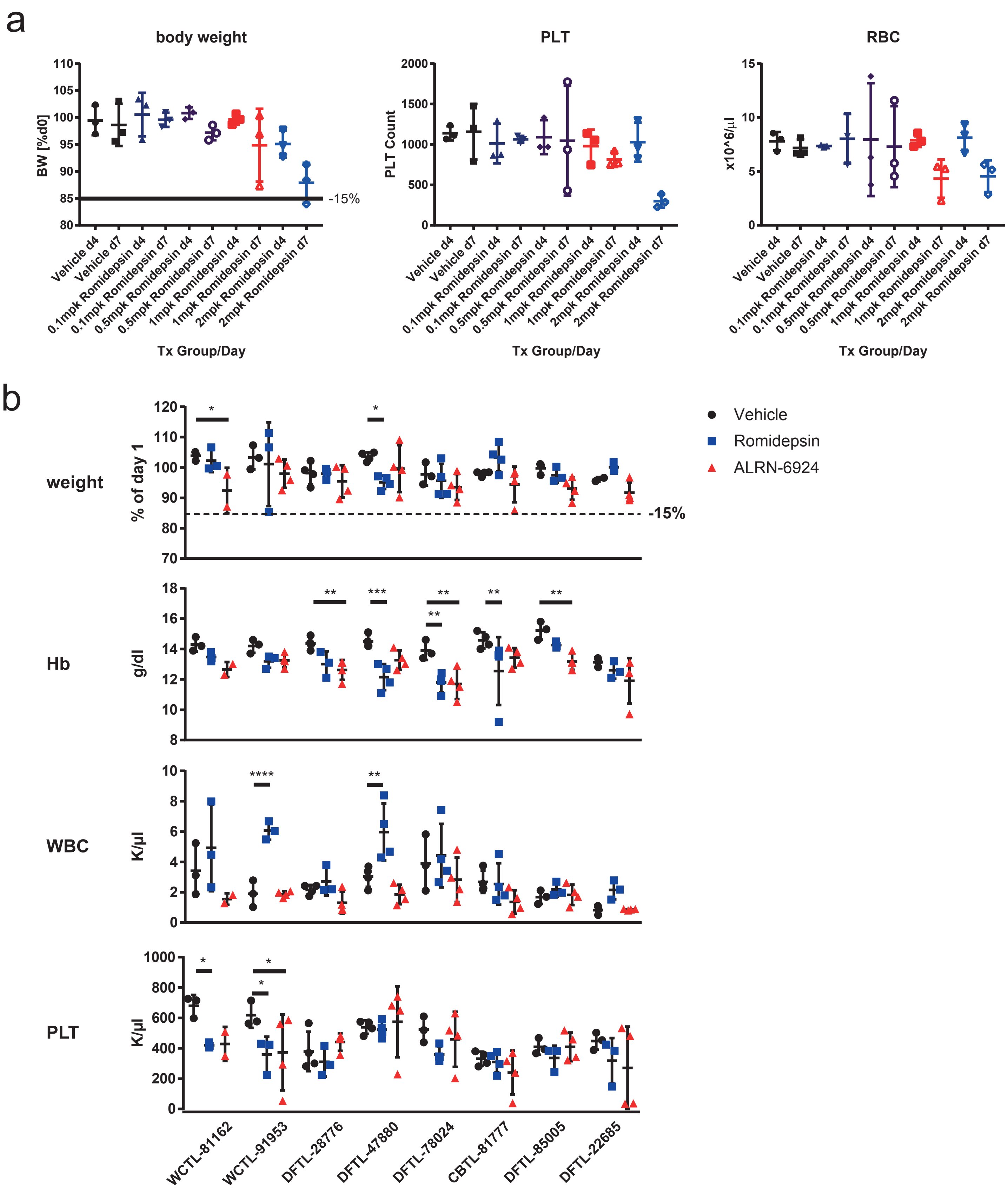

Supplementary Figure 7: MTD and toxicity profiles of ALRN6924 and romidepsin

a) Triplicates of mice engrafted with DFTL-78024 were used to establish a maximum tolerated dose (MTD) of romidepsin given in vivo beginning upon engraftment. Romidepsin 2mg per kg (mpk) given on days 1 and 4 resulted in nearly 15% body weight loss by day 7 as well as reduced platelet (PLT) and red blood cell counts (RBC), and thus was selected as MTD.

b) Toxicity profile of romidepsin and ALRN-6924 in vivo by body weight, hemoglobin (Hb), white blood cell counts (WBC) and platelet counts (PLT). Comparisons are by 2-way ANOVA and Bonferroni correction.

Data points in a) and b) are mean values with error bars indicating standard error of the mean.

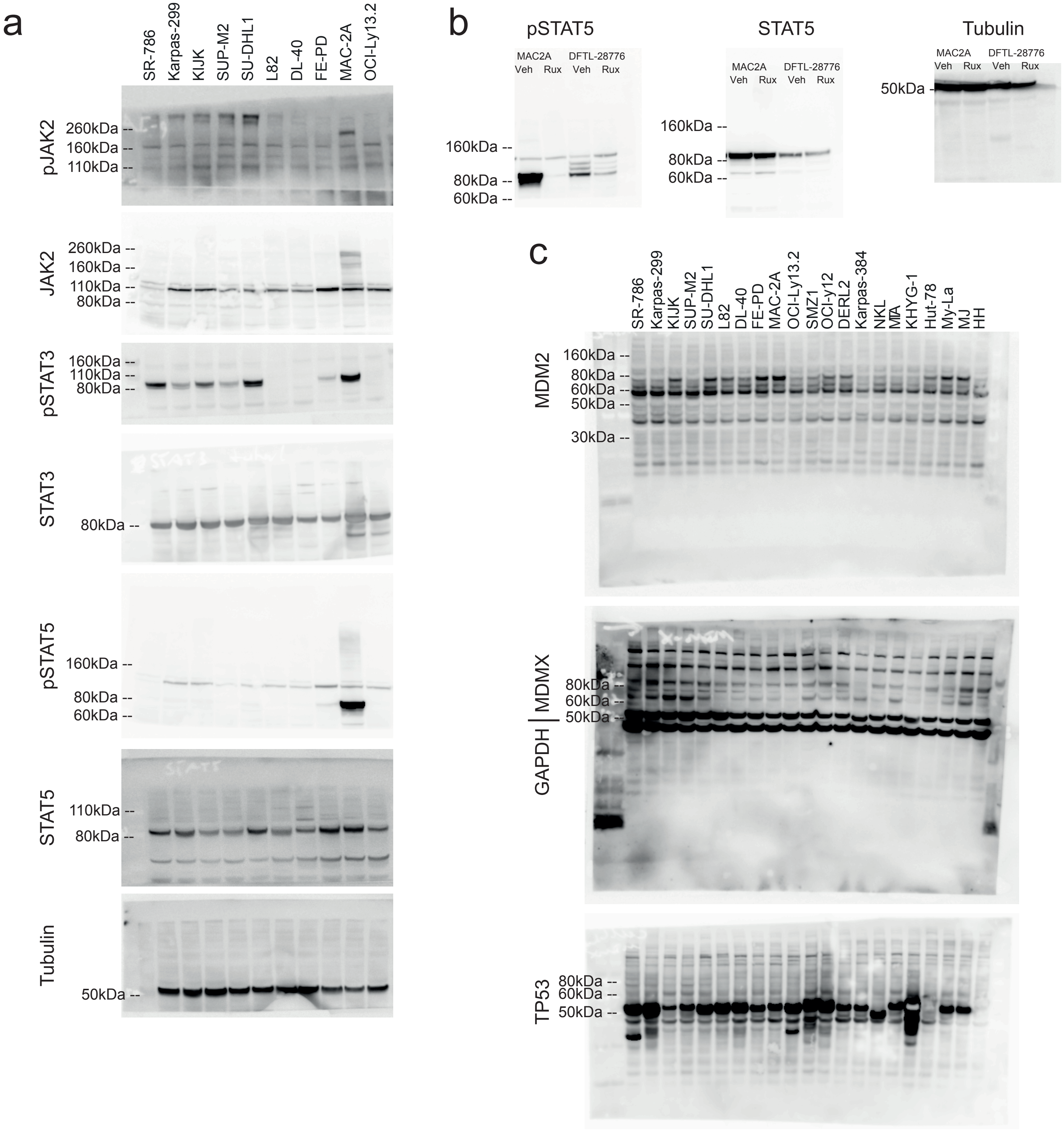

Supplementary Figure 8: Uncropped western blots

a) Original western blot of Figure 1 d

b) Original western blots of Figure 1 i

c) Original western blots of Figure 3 b

**Classification**	**Cell line**	**PDX**
T-cell prolymphocytic leukemia		DFTL-28776, DFTL-20763
T-cell large granular lymphocytic leukemia	MOTN-1	
Chronic lymphoproliferative disorder of NK cells	NKL	
Aggressive NK-cell leukemia	MTA, KHYG-1	
Systemic EBV+ T-cell lymphoma of childhood		
Hydroa vacciniforme–like lymphoproliferative disorder		
Adult T-cell leukemia/lymphoma	ST1, SU9T01, KOB, MT2, MT4, HUT 102	DFTL-69579*
Extranodal NK-/T-cell lymphoma (nasal)		DFTL-85005*
Enteropathy-associated T-cell lymphoma		
Monomorphic epitheliotropic intestinal T-cell lymphoma		
Indolent T-cell lymphoproliferative disorder of the GI tract		
Hepatosplenic T-cell lymphoma	DERL-2	CBTL-81777
subcutaneous panniculities-like T-cell lymphoma		
Mycosis fungoides	My-La, MJ	
Sézary syndrome	HUT 78, H9, Se-Ax	DFTL-90501*, DFTL-58239
Primary cutaneous CD30+ T-cell lymphoproliferative disorders		DFTL-22685
Lymphomatoid papulosis		
Primary cutaneous anaplastic large cell lymphoma	HH	
Primary cutaneous γδ T-cell lymphoma	Karpas-384	
Primary cutaneous CD8+ aggressive epidermotropic cytotoxic T-cell lymphoma		
Primary cutaneous acral CD8+ T-cell lymphoma		
Primary cutaneous CD4+ small/medium T-cell lymphoproliferative disorder		
Peripheral T-cell lymphoma, NOS	SMZ1, OCI-Ly12	DFTL-35806, DFTL-82248
Angioimmunoblastic T-cell lymphoma		DFTL-94393*, DFTL-78024, DFTL-47880, DFTL-75418
Follicular T-cell lymphoma		
Nodal peripheral T-cell lymphoma with TFH phenotype		
Anaplastic large-cell lymphoma, ALK+	SR-786, Karpas-299, Ki-JK, SUP-M2, SU-DHL-1, L82	WCTL-81162, WCTL-91953
Anaplastic large-cell lymphoma, ALK-	DL-40, FEPD, Mac-2A, OCI-Ly13.2	
Breast implant-associated anaplastic large-cell lymphoma	TLBR-1, TLBR-2, TLBR3	DFTL-51398*

Supplementary Table 1: Cell lines and PDX models according to the 2016 revision of the World Health Organization classification of mature T- and NK-cell lymphomas. Provisional entities are printed in *italics* . Previously published PDX models are indicated by * and published in Townsend et al.[4]

Name	Subtype	Immunophenotype	STR profile										Ref.
			TH01	D21S11	D5S818	D13S317	D7S820	D16S539	CSF1PO	AMEL	vWA	TPOX	
SR786	ALK+ ALCL	CD2$^-$ CD3$^-$ CD4$^+$ CD5$^-$ CD7$^-$ CD8$^-$ CD30$^+$	6,7	28,30	11	11,13	9,12	11,13	14,15	X	17,18	8,11	32
Karpas-299	ALK+ ALCL	CD2$^-$ CD3$^-$ CD4$^+$ CD5$^+$ CD7$^-$ CD8$^-$ CD30$^+$	7	27,29	11,12	8,12	10,11	12	10,11	X,Y	17,19	8	39
KIJK	ALK+ ALCL	CD2$^-$ CD3$^-$ CD4$^-$ CD5$^+$ CD7$^+$ CD8$^-$ CD30$^+$	8,9	29	11,12	9,12	10,12	10	12,13	X,Y	17,18	10,11	33
SUPM2	ALK+ ALCL	CD2$^+$ CD3$^-$ CD4$^+$ CD5$^-$ CD7$^-$ CD8$^-$ CD30$^+$	8,9.3	29,30	11	10,11	8,12	11,12	11,12	X	16,18	8,10	34
SUDHL1	ALK+ ALCL	CD2$^-$ CD3$^-$ CD4$^-$ CD5$^+$ CD7$^-$ CD8$^-$ CD30^{lo}	6,7	29,30	11,12	9,13	10,13	11,12	12	X,Y	15,17	8	35
L82	ALK+ ALCL	CD2$^-$ CD3$^-$ CD4$^+$ CD5$^+$ CD7$^-$ CD8$^-$ CD30$^+$	9,3	31.2,33.2	12	8,12	10,11,12	10,11	10,12	X	16,17	8,11	36
WCTL-81162	ALK+ ALCL	CD2$^-$ CD3$^-$ CD4^{dim} CD5$^+$ CD7^{dim} CD8$^-$ CD30+	7,10	31,2	9,10	8,10	8,11	11,13	11,12	X	16,17	8,11	
WCTL-91953	ALK+ ALCL	CD2$^-$ CD3$^-$ CD4$^-$ CD5$^-$ CD7$^+$ CD8$^-$ CD30+	6,9.3	29,30	11,13	11	9	8,11	11,13	X,Y	16,17	8	
DFTL-51398	ALK- ALCL (TLBR)	CD2$^-$ CD3$^-$ CD4$^-$ CD5$^-$ CD7$^+$ CD8$^-$	9,9.3	29,32.2	10,11	12	8,9	11,12	11,12	X	14,16	8,11	4
DL-40	Alk- ALCL	CD2$^+$ CD3$^-$ CD4$^+$ CD5$^-$ CD7$^-$ CD8$^-$ CD30$^+$	8	30,31	9,12	9,13	10,11	10,11	13,14	X	14,19	8,9	44
FEPD	Alk- ALCL	CD2$^+$ CD3$^-$ CD4$^+$ CD5$^-$ CD7$^-$ CD8$^-$ CD30$^+$	9,3	28,31	12	11,12	10	9,13	11,12	X	17,18	8	49
MAC2A	Alk- ALCL	CD2$^-$ CD3$^-$ CD4$^-$ CD5$^-$ CD7$^-$ CD8$^-$ CD30$^+$	8,9.3	28,30.2	11,12	8,12	10	11,13	11,12	X	14,18	8	50
OCI-Ly13.2	Alk- ALCL	CD2$^+$ CD3$^+$ CD4$^-$ CD5$^-$ CD7$^+$ CD8$^-$ CD30$^+$	7	30,30.2	12,13	11	10	11,13	9,10	X	17	11	51
SMZ1	PTCL-NOS	CD2$^+$ CD3$^+$ CD4$^+$ CD5^{lo} CD7^{lo} CD8$^-$ CD30^{lo}	9,9.3	29,31.2	10,13	10,12	8,11	9	11,12	X,Y	16,17	8	60
OCI-Ly12	PTCL-NOS	CD2$^+$ CD3$^-$ CD4$^-$ CD5$^-$ CD7$^+$ CD8$^{(+)}$ CD10$^+$ CD30$^+$	7,8	30,31.2	12,16	11	10,12	12	10,12	X	15,16	8	51
DFTL-35806	PTCL-NOS	CD$^+$ CD4$^+$ CD7^{sub} CD30dim	8,9.3	27,28	12	12,13	8,12	9,12	10	X,Y	17,18	8	
DFTL-82248	PTCL-NOS	CD3$^+$ CD4^{sub} CD7^{sub} CD8$^+$	7,9	29	11	13,14	11,13	11	10,13	X,Y	16	8,9	
DFTL-78024	AITL	CD2$^+$ CD3$^+$ CD4$^+$ CD5$^+$ CD8$^-$ PD1$^+$	7,9	29	10,12	8	10,11	9,10	10,13	X,Y	14,18	9,11	
DFTL-47880	AITL	CD2$^+$ CD3$^+$ CD4$^+$ CD5$^+$ CD8$^-$ PD1$^+$	9,3	28,30	12,13	11,12	10,13	9,12	10,12	X,Y	16,18	8,9	
DFTL-94393	AITL	CD2$^+$ CD3$^+$ CD4$^+$ CD5$^+$ CD7$^-$ CD8$^-$	7,8	30,31	12	12,13	10	9,12	11	X,Y	17	8	4
DFTL-75418	AITL	CD2$^+$ CD3$^+$ CD4$^+$ CD5$^+$ CD7$^-$ CD8$^-$	9,9.3	27,29	12,13	9	10,12	10,12	11,12	X,Y	16,18	9	
DERL-2	HS-TCL	CD2$^+$ CD3^{lo} CD4$^-$ CD5$^-$ CD7$^+$ CD8$^+$ CD30$^-$ CD56$^+$	9	29,30	10,11	11,12	11,12	10,11	11,12	X,Y	17,19	8,11	37
CBTL-81777	HS-TCL	CD2$^+$ CD3$^+$ CD4$^-$ CD5$^-$ CD7$^+$ CD8$^+$	7,8	30,31	12	12,13	10	9,12	11	X,Y	17	8	
Karpas-384	Subcutaneous yδ TCL	CD2$^-$ CD3$^-$ CD4$^-$ CD5$^-$ CD7$^-$ CD30$^-$ cyCD3$^+$	7,9	31,32	11	10,11	13	11,12	10	X,Y	15,17	8	40
NKL	NKT	CD2$^+$ CD3$^-$ CD4$^-$ CD5$^-$ CD7$^-$ CD8$^-$ CD56^{dim}	7,8	29,30	12	11,12	9,10	10,13	10,12	X,Y	18	8	52
MTA	NKL	CD2$^+$ CD3$^+$ CD4$^+$ CD5$^-$ CD7$^-$ CD28$^+$ CD56$^+$	6,7	29,30	10,11	11	10,11	9,12	10,11	X	18,19	8,11	45
KHYG-1	NKL	CD2$^+$ CD3$^-$ CD4$^-$ CD5$^-$ CD7$^+$ CD8$^+$ CD56$^+$	6	30,32.2	9,11	9,13	10,11	9	12	X	17	9,11	46
DFTL-85005	NKT	CD3$^-$ CD4$^-$ CD8$^-$ CD16$^-$ CD56$^+$ CD57$^-$	7,9.3	30,31.2	12	11,12	9,12	13,14	10,11	X,Y	14,15	8	4
HUT78	CTCL	CD2$^+$ CD3$^+$ CD4$^+$ CD5$^+$ CD7$^+$ CD8$^-$	8,9	30	11	8,12	8,11	11,12	11,12	X,Y	14,15	8,9	42
MyLa	CTCL	CD2$^+$ CD3$^+$ CD4$^-$ CD8$^+$	6,6	28,33.2	9,12	11,12	10,11	8,11	11,12	X,Y	17	11,12	41
MJ	CTCL	CD2$^+$ CD3$^+$ CD4$^+$ CD8$^-$	6,7	28,31.2	11,13	12,14	8,11	11,12	11,12	X,Y	15,17	8,11	43
HH	CTCL	CD2$^+$ CD3$^+$ CD4$^+$ CD5$^+$ CD7$^-$ CD8$^-$CD30+	9	30,32.2	11,13	11,12	10	12	11,12	X	15,16	8,11	53
H9	CTCL	CD2$^+$ CD3$^+$ CD4$^+$ CD5$^+$ CD7$^+$ CD8$^-$	8,9	30	11	8,12	8	11,12	11	X,Y	14,15	8,9	42
SeAx	CTCL	CD2$^+$ CD3$^+$ CD4$^-$ CD5$^-$ CD8$^-$	9,3	28,31	12,13	12,14	7	9	11,12	X	16	11	54
DFTL-22685	CTCL	cyCD3$^+$ CD4$^+$ CD5$^+$ CD8$^-$	7,8	28,29	10,11	11,12	9,12	9,12	7,12	X,Y	15	9,11	
DFTL-58239	CTCL	CD3$^+$ CD4^{sub} CD8$^+$ CD30^{sub}	9,3	29,32.2	11,12	12,13	8,11	12,13	12,13	X	17	8,9	4
DFTL-90501	CTCL	CD3$^+$ CD4$^+$ CD7$^-$ CD8$^-$	7,8	28,31	11,13	10,12	11,12	11	11,13	X,Y	17,18	11	4
MOTN1	T-LGL	CD2$^+$ CD3$^-$ CD4$^+$ CD5$^-$ CD7$^+$ CD8$^-$	6	30,31	10	11,12	10,12	9,11	12,13	X	14,19	11	38
HUT102	ATLL	CD2$^+$ CD3$^-$ CD4$^+$ CD5$^+$ CD7$^-$ CD8$^-$	7,8	28,29	8,13	11,13	8,10	12	8,11	X,Y	16,19	6	55
KOB	ATLL	CD2$^+$ CD3$^-$ CD4$^+$ CD5$^+$ CD7$^-$ CD8$^-$	6,9	32,2	12,13	8,11	9,11	9,11	11,12	X	16,18	9	58
MT2	ATLL	CD2$^+$ CD3$^-$ CD4$^+$ CD5$^-$ CD7$^-$ CD8$^-$	6,10	29,30	13	9,13	8,13	9,11	10,11	X,Y	16	10,11	56
MT4	ATLL	CD2$^+$ CD3$^-$ CD4$^+$ CD5$^+$ CD7$^+$ CD8$^+$	7	28	10,11	12	8,10	9,12	11,12	X,Y	17,18	11	57
ST1	ATLL	CD2$^+$ CD3$^+$ CD4$^-$ CD5$^+$ CD7$^+$ CD8$^+$	6,9	30,32.2	13	9,11	11	9,10	10,11	X	14,19,20	11	58
Su9T01	ATLL	CD2$^+$ CD3$^+$ CD4$^-$ CD5$^-$ CD7$^+$ CD8$^+$	7,8	30,32.2	10	10	8,11	12	10,11	X,Y	17,21	9	59
DFTL-69579	ATLL	CD2$^+$ CD3$^+$ CD4$^+$ CD5$^+$ CD7$^-$ CD8^{sub}	7,9	28,31.2	11,12	10,12	9,10	11,13	8,13	X	16,18	6,11	4
DFTL-20763	T-PLL	CD3$^+$ CD4$^+$ CD7^{dim} CD8$^-$ CD10$^-$	7,9	28, 34.2	10,12	12,13	8,9	9,11	10,11	X	16,20	7,8	
DFTL-28776	T-PLL	cyCD3$^+$ CD4$^+$ CD5$^+$ CD7$^+$ CD8$^-$	9,9.3	28,32.2	9,11	11,12	10,11	11,12	11,12	X	16,17	8,11	

Supplementary Table 2: Immunophenotype, STR profiles and references for TCL lines. Cell lines and PDX models of ALCL typically lack surface expression of CD3 and in many cases CD2 and CD4, are consistently negative for CD8 but express high levels of CD30. In contrast, the PTCL-NOS cell lines SMZ-1 and OCI-Ly12 reflect the heterogeneity of this subtype with expression of CD2, CD3, and CD4, but loss of CD5 and CD7 and low expression of CD30 in SMZ1 but loss of CD3, CD4 and CD5 in OCI-Ly12 with expression of CD7, partial expression of CD8 and strong CD30. CTCL cell lines of Sézary Sydrome and Mycosis fungoides are CD3 and CD4 positive. However, MyLa is CD4- CD8+. Strong CD30 expression of the cell line HH is consistent with large cell-transformation of the patient's initial Sézary Syndrome.

Subtype	WGS / WES	Targeted Sequencing	Reference
ALK+ ALCL	7		Crescenzo et al., Cancer Cell 2015
		4	Palomero et al., Nature Genetics 2014
Alk- ALCL	16		Crescenzo et al., Cancer Cell 2015
		2	Palomero et al., Nature Genetics 2014
PTCL-NOS	6	25	Palomero et al., Nature Genetics 2014
	3		Sakata-Yanagimoto et al., Nature Genetics 2014
	5		Yoo et al., Nature Genetics 2014
		28	Schatz et al., Leukemia 2015
AITL	3	30	Palomero et al., Nature Genetics 2014
	3		Sakata-Yanagimoto et al., Nature Genetics 2014
		85	Odejide et al., Blood 2014
HS-TCL	68		McKinney et al., Cancer Discovery 2017
NKT	2		Palomero et al., Nature Genetics 2014
	25	80	Jiang et al., Nature Genetics 2015
CTCL	201	10	Park et al., Blood 2017
T-PLL	40		Kiel et al., Blood 2014
ATLL	83		Kataoka et al., Nature Genetics 2015
		41	Nakagawa et al., JEM 2014
		1	Palomero et al., Nature Genetics 2014
EATL	69		Moffitt et al., JEM 2017
	1		Palomero et al., Nature Genetics 2014
T-LGL	19		Coppe et al., Leukemia 2017

Supplementary Table 3, Reported genetic characterization of patient T-cell lymphomas. Number of cases from reported sequencing studies, included in Figure S2. WGS/WES, whole genome sequencing/whole exome sequencing.

ABL1	CD58	EPHA7	IDH2	MUC17	PHF6	SEMA3A	TET2
ADAMTS9	CDH10	ETS1	IKZF1	MUC2	PHIP	SENP6	THSD7A
AIM1	CDK4	ETV6	IKZF2	MUC5B	PIK3AP1	SETBP1	TLR3
AKT3	CDK6	EZH2	IKZF3	MUM1	PIK3CA	SETD2	TNFAIP3
ALK	CDKN2A	FAM75D1	IL6R	MYC	PIK3CD	SF3B1	TNFRSF14
ALMS1	CDKN2B	FAS	IL7R	MYD88	PIK3R1	SH2B3	TNFRSF21
ALPK2	CEBPA	FAT1	IRF4	MYH9	PIM1	SLC8A3	TNFSF9
ALPP	CHST6	FAT4	JAK1	NCKAP5	PKHD1	SLITRK6	TP53
ANKRD50	CHSY3	FBXO11	JAK2	NCOA3	PLCG1	SMARCA2	TP63
APC	CIITA	FBXW7	JAK3	NEFH	PLXNC1	SMARCAL1	TRAF3
ARID1A	CMYA5	FLT3	KBTBD5	NEIL3	PPM1D	SMARCD1	TRAF6
ARID1B	CNOT3	FNBP4	KCNN3	NFKBIA	PRDM1	SMC1A	TTC27
ARID2	CNOT4	FOXO1	KIF7	NLRP7	PRF1	SMC3	TTC28
ARID3A	CRAMP1L	FOXO3	KIT	NOTCH1	PRKD2	SOCS1	TUBGCP6
ASXL1	CREBBP	FYN	KRAS	NOTCH2	PRPF8	SRGAP3	TYK2
ASXL2	CSF1R	GABRR1	LILRB1	NPM1	PTEN	SRSF2	TYRP1
ATG5	CSMD2	GATA3	LMO2	NR5A2	PTPN11	STAB1	U2AF1
ATM	CSMD3	GNA13	LOC283710	NRAS	PTPRD	STAG2	ULK4
B2M	CTCF	GNAQ	LSR	NT5C2	PTPRM	STAT1	UNC80
BACH2	CTNNB1	GNAS	MAGEC1	NT5C3A	RAD21	STAT3	USP8
BANK1	CTTNBP2	GNB1	MALT1	P2RY8	RBMXL2	STAT5A	VAV1
BCL11A	CUL9	GNB2	MAZ	PAPLN	RC3H1	STAT5B	VPS13A
BCL11B	DDX3X	GPD2	MCL1	PARD3	REL	STAT6	WT1
BCL6	DENND1B	GRIK4	MDM2	PASD1	RELN	SUZ12	XIRP2
BCL7A	DMD	HACE1	MEF2B	PASK	RFTN1	SYK	XPO1
BCORL1	DNAH5	HCK	MEF2C	PAX5	RHOA	SYNE1	ZFHX3
BCR	DNM2	HERC1	MET	PCDHA11	RHOT2	TACC2	ZRSR2
CBL	DNMT3A	HIST1H1B	MGAT4C	PCLO	RNF213	TAF1	
CCL2	DST	HIST1H3J	MLL	PCMTD1	RPL10	TAL1	
CCND1	DYNC2H1	HIVEP1	MLL2	PDCD6	RPL5	TATDN2	
CCND2	EED	HLA-DPB1	MLL3	PDE4DIP	RUNX1	TBC1D8B	
CCND3	EIF3A	HMCN1	MPRIP	PDGFC	RYR3	TBL1XR1	
CCR4	EP300	ID3	MPS1	PDGFRA	SAMD9	TCF3	
CD28	EPC1	IDH1	MUC12	PDGFRB	SCAF1	TET1	

Supplementary Table 4: Gene list for targeted exon capture and next-generation sequencing of all coding exons

ARID1A	CD70	HIST1H1C	MEF2B	RHOA
ATM	CD79b	HIST1H1E	KMT2D/MLL	RRAGC
B2M	CDKN2A	HIST1H2BO	MYC	SF3B1
ABL1	CHEK2	HIST1H3G	MYD88	STAT3
ACTB	CREBBP	HIST2H2AC	NOTCH1	STAT5b
ATP6AP1	CTCF	HNRNPAB1	NOTCH2	STAT6
ATP6V1B2	CXCR4	ID3	NRAS	SUZ12
BCL2	DDX3X	IDH1	NT5C2	TCF3
BIRC3	DNMT3A	IDH2	P2RY8	TET2
BRAF	DTX1	IL2RG	PARP1	TNFAIP3
BTG1	ERG	IL7R	PHF6	TNFRSF14
BTG2	ETS1	IRF4	PIM1	TP53
BTK	EZH2	IRF8	PLCG1	TRAF2
CARD11	FAS	JAK1	PLCG2	TRAF3
CCND1	FBXW7	JAK2	POU2F2	VAV1
CCND3	FOXO1	JAK3	PRDM1	WHSC1
CCR4	FOXP1	KLHL6	PRKCB	XPO1
CCR7	FYN	KRAS	PTEN	
CD28	GATA3	LYN	PTPN11	
CD58	GNA13	MAP2K1	RB1	

Supplementary Table 5: List of target genes of the Rapid Lymphoma Panel

Gene	Exons	Gene	Exons	Gene	Exons	Gene	Exons
ABL1	1-11	ETV6	1-6	MKL1	4-6	RAB7A	3,5
ABL2	2-8	FGFR1	2-12,17	NOTCH1	24-29,34	RARA	1-9
ALK	2,10,16-23,25	FLT3	14-18,20	NRAS	2-3	RBM15	1
BCR	1-3,8,12-16	GPI	12,16	NTRK3	4,7,10,13-16	RUNX1	1-9
BRAF	15	IKZF1	1-3,7-8	NUP214	17-19	RUNX1T1	1-9
CBFB	4,5	IL7R	5-6	NUP98	8-17	SEMA6A	1-2
CHD1	1-2	JAK1	14-16	P2RY8	1	SETD2	1-12
CHMP2A	3,5	JAK2	6-13,15-20,22	PAX5	1,4-8	SH2B3	2
CRLF2	1-6	JAK3	11,13,15,18	PDGFRA	9-14,18	TAL1	2-6
CSF1R	9-14	KLF2	2-3	PDGFRB	8-14	TCF3	11-18
EBF1	10-15	KMT2A	2-35	PICALM	16-19	TYK2	16,18
EPOR	7-8	KRAS	2-4	PTK2B	2-8	ZCCHC7	1-4
ERG	7-11	MECOM	1-5,8	PTPN11	3,13		

Supplementary Table 6: List of target genes of the Archer Fusion Panel
