# Supplementary material for: Targetable vulnerabilities in T- and NK-cell lymphomas identified through preclinical models
Source: Nat Commun. 2018 May 22;9:2024. doi: 10.1038/s41467-018-04356-9 (PMC5964252; doi:10.1038/s41467-018-04356-9)
Supplement: Supplementary file 3 — Description of Additional Supplementary Files [file 41467_2018_4356_MOESM3_ESM.pdf]

Description of Additional Supplementary Files

File Name: Supplementary Data 1
Description: Copy number variants called by the GISTIC algorithm

File Name: Supplementary Data 2
Description: Fusions called by RNA-Seq in cell lines

File Name: Supplementary Data 3
Description: GSEA of Alk+ ALCL vs all other subtypes, according to clustering in Supplementary Figure 4a

File Name: Supplementary Data 4
Description: GSEA of PDX models of AITL vs PDX models of other subtypes in Supplementary Figure 4a

File Name: Supplementary Data 5
Description: Avana_CERES_Dependency Scores (ZMAD) across the 8 TCL lines

File Name: Supplementary Data 6
Description: Avana_CERES_FDR of dependency across the 8 TCL lines

File Name: Supplementary Data 7
Description: CNV cell lines, gene level

File Name: Supplementary Data 8
Description: Mutations cell lines

File Name: Supplementary Data 9
Description: Mutations PDXs

File Name: Supplementary Data 10
Description: RNASeq gene level
