# Supplementary material for: Targetable vulnerabilities in T- and NK-cell lymphomas identified through preclinical models
Source: Nat Commun. 2018 May 22;9:2024. doi: 10.1038/s41467-018-04356-9 (PMC5964252; doi:10.1038/s41467-018-04356-9)
Supplement: Supplementary file 2 — Peer Review File [file 41467_2018_4356_MOESM2_ESM.pdf]

Editorial note: This manuscript has been previously reviewed at another journal that is not operating a transparent peer review scheme. This document only contains reviewer comments and rebuttal letters for versions considered at Nature Communications.

REVIEWERS' COMMENTS:

Editorial note: Reviewer 2 commented for the editors only:

This reviewer suggests that if it is suggested that the drugs are metabolised differently across species, they authors should cite the work supporting this statement and describe how this was kept into consideration in the analysis, in the discussion or in the methods section.

Reviewer #3 (Remarks to the Author):

The manuscript by Ng and coworkers characterizes preclinical models (cell lines and primary sample derived xenografts) of peripheral T cell lymphomas and integrates these results with analysis of vulnerabilities identified via genome wide CRISPR screens. Based on these results preclinical testing of a staple peptide inhibitor of MDM2 and MDMX is evaluated in favor of the relevance of these models and approaches. The Peripheral T-cell lymphoma category encompasses different diseases emerging from different cellular compartments and characterized by distinct molecular profiles. The studies presented here aim to address the limitations imposed by the lack of adequate models and represent a substantial effort towards the identification of druggable vulnerabilities. In this, the manuscript provides: (I) a resource paper providing data and new resources to the community and (II) a discovery manuscript reporting novel genetic alterations and therapeutic targets. However in its current stage these represent a moderate advance. As a resource manuscript the diversity of models analyzed is not comprehensive, particularly regarding the CRISPR screen. Regarding the identification of new therapeutic targets, there is limited mechanistic novel insight and much of the results are either validation-extension of previous observations or have an unclear path towards clinical application. Altogether, this work is better suited for publication in a specialized journal.
Specific comments:
“We identify multiple vulnerabilities that are targetable with currently available agents (e.g. inhibitors of JAK2, PDGFRB, or IKZF1) and demonstrate proof-of-principle for biomarker-driven therapies using patient-derived xenografts (PDXs).”: It is unclear that the PDGFB alteration identified here is a driver oncogenic druggable target.
Extended data table 5 lists over 1200 fusion calls in cell lines: it is unclear how driver gene fusions were further selected.
“To identify copy number alterations, we applied the GISTIC (Genomic Identification of Significant Targets in Cancer) algorithm to whole exome sequencing data from 20 cell lines.” Lack of xenograft and primary sample data limits the impact of these analyses.
“A preliminary analysis demonstrated lower IRF4 protein levels in lines that were sensitive to lenalidomide (p=0.0025; Extended Data Figure 5c,d).” As indicated before this is unconvincing. Qualifying the results as “preliminary” does not make them any more valid.
Validation of PTPN2 vulnerability is a nice addition. Yet the mechanism and venue for targeting remain unclear.
Micrograph panels for p53 and GAPDH are missing in Extended data figure 6c
Micrograph on ALRN-6924 WCTL-91953 is missing in Extended data figure 6i
There is no human subjects section in the Methods. Reporting of an exceptional response from a single patient is not consistent with the standards for publication of clinical trial results.

REVIEWERS' COMMENTS:

Editorial note: Reviewer 2 commented for the editors only:

This reviewer suggests that if it is suggested that the drugs are metabolised differently across species, they authors should cite the work supporting this statement and describe how this was kept into consideration in the analysis, in the discussion or in the methods section.

We appreciate the Reviewer’s point. The drugs we utilized (like essentially all pharmacologic agents) are undoubtedly metabolized differently across species. We’ve removed any statements that would require referencing, as this is a well-known point.

Reviewer #3 (Remarks to the Author):

The manuscript by Ng and coworkers characterizes preclinical models (cell lines and primary sample derived xenografts) of peripheral T cell lymphomas and integrates these results with analysis of vulnerabilities identified via genome wide CRISPR screens. Based on these results preclinical testing of a staple peptide inhibitor of MDM2 and MDMX is evaluated in favor of the relevance of these models and approaches. The Peripheral T-cell lymphoma category encompasses different diseases emerging from different cellular compartments and characterized by distinct molecular profiles. The studies presented here aim to address the limitations imposed by the lack of adequate models and represent a substantial effort towards the identification of druggable vulnerabilities. In this, the manuscript provides: (I) a resource paper providing data and new resources to the community and (II) a discovery manuscript reporting novel genetic alterations and therapeutic targets. However, in its current stage these represent a moderate advance. As a resource manuscript the diversity of models analyzed is not comprehensive, particularly regarding the CRISPR screen. Regarding the identification of new therapeutic targets, there is limited mechanistic novel insight and much of the results are either validation-extension of previous observations or have an unclear path towards clinical application. Altogether, this work is better suited for publication in a specialized journal.

We are pleased that the cumulative impressions of the other Reviewers outweighed the final sentence of the previous paragraph from this single reviewer. We feel strongly that *Nature Communications* is an ideal location for this manuscript, as it will offer open access to a broad swatch of researchers.

Specific comments:
“We identify multiple vulnerabilities that are targetable with currently available agents (e.g. inhibitors of JAK2, PDGFRB, or IKZF1) and demonstrate proof-of-principle for biomarker-driven therapies using patient-derived xenografts (PDXs).”: It is unclear that the PDGFB alteration identified here is a driver oncogenic druggable target.

We have removed PDGFRB from this statement.

Extended data table 5 lists over 1200 fusion calls in cell lines: it is unclear how driver gene fusions were further selected.

We selected fusions with the strongest evidence, including read count >50, and then manually curated for fusions involving kinase or other domains that are potentially targetable with current agents. We have made clear in the text that we show only a selection of potentially targetable fusions in Figure 1.

“To identify copy number alterations, we applied the GISTIC (Genomic Identification of Significant Targets in Cancer) algorithm to whole exome sequencing data from 20 cell lines.” Lack of xenograft and primary sample data limits the impact of these analyses.

We agree but extensive analysis of primary samples has been published for multiple PTCL subsets and is underway for others.

"A preliminary analysis demonstrated lower IRF4 protein levels in lines that were sensitive to lenalidomide (p=0.0025; Extended Data Figure 5c,d)." As indicated before this is unconvincing. Qualifying the results as "preliminary" does not make them any more valid.

The Reviewer is not clear whether he/she prefers us to take the analysis out or to somehow further modify the language. Unfortunately, those are the only two choices with the available data. We're amenable to either but believe that the analysis is valid (i.e. the measurements are accurate and the statistics are appropriate). We have attempted to convey that this is simply a comparison across cell lines and that further data is needed, but it also represents a statistically robust observation that we believe merits reporting (rather than burying).

Validation of PTPN2 vulnerability is a nice addition. Yet the mechanism and venue for targeting remain unclear.

We agree completely.

Micrograph panels for p53 and GAPDH are missing in Extended data figure 6c

Corrected as requested.

Micrograph on ALRN-6924 WCTL-91953 is missing in Extended data figure 6i

Corrected as requested.

There is no human subjects section in the Methods. Reporting of an exceptional response from a single patient is not consistent with the standards for publication of clinical trial results.

We have added a human subjects section and are clear that we are reporting only a single patient and not a clinical trial experience, which needs to be completed and reported separately.
